# Degradable and Reprocessable Resins from a Dioxolanone Cross-Linker

**DOI:** 10.1021/acs.macromol.2c02560

**Published:** 2023-02-09

**Authors:** Theona Şucu, Meng Wang, Michael P. Shaver

**Affiliations:** † Department of Materials, Engineering Building A, University of Manchester, Oxford Road M13 9PL, U.K.; ‡ Sustainable Materials Innovation Hub, Henry Royce Institute, University of Manchester, Manchester M13 9PL, U.K.

## Abstract

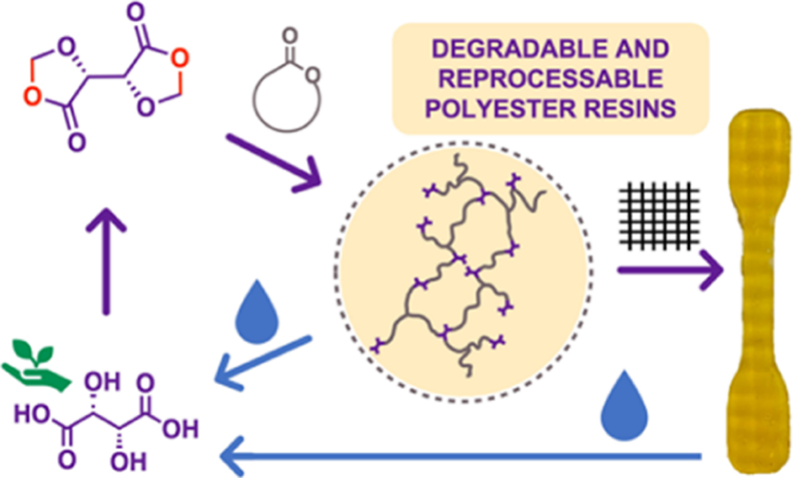

Chemically cross-linked polymers offer excellent temperature and solvent resistance, but their high dimensional stability precludes reprocessing. The renewed demand for sustainable and circular polymers from public, industry, and government stakeholders has increased research into recycling thermoplastics, but thermosets have often been overlooked. To address this need for more sustainable thermosets, we have developed a novel bis(1,3-dioxolan-4-one) monomer, derived from the naturally occurring l-(+)-tartaric acid. This compound can be used as a cross-linker and copolymerized *in situ* with common cyclic esters such as l-lactide, ε-caprolactone, and δ-valerolactone to produce cross-linked, degradable polymers. The structure–property relationships and the final network properties were tuned by both co-monomer choice and composition, with properties ranging from resilient solids with tensile strengths of 46.7 MPa to elastomers with elongations up to 147%. In addition to exhibiting properties rivalling those of commercial thermosets, the synthesized resins could be recovered at end-of-life through triggered degradation or reprocessing. Accelerated hydrolysis experiments showed the materials fully degraded to tartaric acid and the corresponding oligomers from 1 to 14 days under mild basic conditions and in a matter of minutes in the presence of a transesterification catalyst. The vitrimeric reprocessing of networks was demonstrated at elevated temperatures, and rates could be tuned by modifying the concentration of the residual catalyst. This work develops new thermosets, and indeed their glass fiber composites, with an unprecedented ability to tune degradability and high performance by creating resins from sustainable monomers and a bio-derived cross-linker.

## Introduction

The thermoset industry has grown significantly, with cross-linked polymers now ubiquitous in everyday life, constituting 15–20% of the polymers produced.^1−3^ These resins offer additional dimensional stability, enhanced tensile and impact strengths, improved elasticity, and reduced creep, making them essential for use as insulators, adhesives, coatings, foams, and automotive parts.^4−8^ Although thermoplastics benefit from multiple end-of-life pathways, such as mechanical recycling, chemical recycling, or composting, thermosets are mostly incinerated or landfilled.^9−11^ While circularizing linear polymers remains at the forefront of academic research,^12−14^ innovation regarding thermoset circularity lags behind due to their inherent resilience. Their permanent architecture precludes flow, even at high temperature, so traditional reprocessing such as mechanical recycling is elusive. Moreover, due to their insolubility, solution reprocessing is also impeded.^15^ The lack of traditional waste management streams, coupled with the higher value potential applications, suggests that design for degradability or improved reprocessing could open more sustainable fates for cross-linked polymers at end of life.

While most thermosets do not have labile bonds, cross-linked networks containing polyester backbones are some of the most widely employed and commercially relevant thermosets with potential for circularity.^2^ Degradation of the labile ester linkages could enable recovery of the starting materials and/or formation of different small molecules that can be readily metabolized.^16−18^ However, a key consideration in the pursuit of more sustainable materials (where environmental, social, and economic sustainability must all be considered) is the need for design to be informed by practice, rather than practice changed by design.^19^ It is imperative that the polymers created have an assured fate, meaning both degradation and recycling pathways need to work for any proposed system.

**Figure 1:**
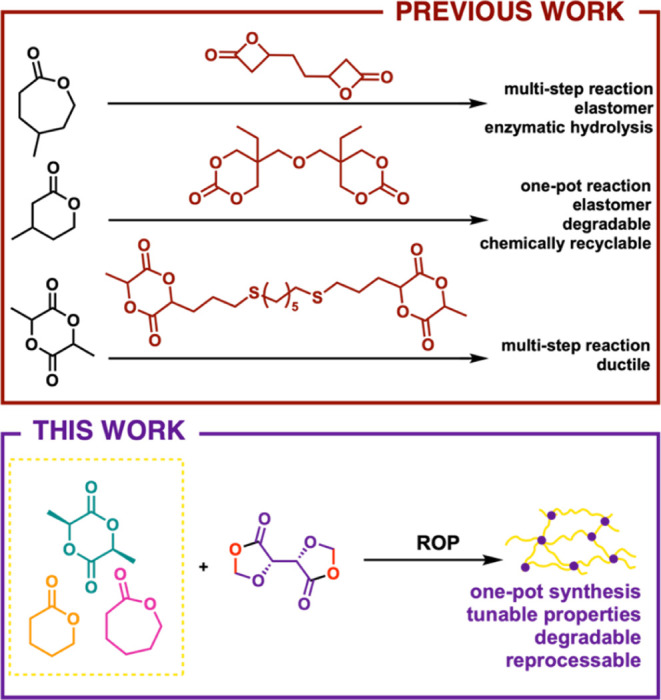
Previous reports of cross-linked polyester synthesis (top) contrasted with the synthesis of cross-linked polyesters using a bis(1,3-dioxolan-4-one) and a traditional cyclic ester co-monomer (bottom).

Economic sustainability necessitates that sustainable thermosets must match or exceed the performance of their traditional counterparts while remaining cost-competitive.^20^ The majority of the reported cross-linked polyesters explored as biodegradable thermosets exhibit mechanical properties that are often limited to low moduli and high elasticity.^21^ A bis-lactide monomer was used by Dove and co-workers (Figure 1, previous work) to synthesize poly(lactic acid) (PLA) thermosets with higher elongation at break but with reduced Young’s modulus and tensile strength compared to the analogous homopolymer.^22^ Bis(cyclic carbonate)s were also reportedly used as cross-linkers to synthesize degradable poly(β-methyl-δ-valerolactone) resins (Figure 1, previous work), whose mechanical properties resembled those of conventional vulcanized rubber. These networks were chemically recyclable and degradable in an acidic environment.^23^ Similar properties were reported by the same group when using a bifunctional bis(β-lactone) to cross-link star-shaped poly(γ-methyl-ε-caprolactone). This time, full enzymatic degradation was observed across a range of temperatures (Figure 1, previous work, top example).^24^ Direct reprocessing, elusive for traditional thermosets, was explored successfully by employing a diisocyanate cross-linker for star-shaped PLA.^25^ In this case, Sn(Oct)_2_ acted as a catalyst for both cross-linking and reprocessing, allowing for rigid materials to be synthesized and reprocessed with full recovery in their tensile strength. The properties of these thermosets make them better suited for applications calling for high flexibility, while the challenging material requirements needed for materials that meet the demands of built environment applications remain elusive. In order to access sustainable resins with high dimensional stability, we sought to explore new cross-linking motifs.

Our group has previously developed a family of 1,3-dioxolan-4-one (DOX) monomers that are easily prepared from renewable α-hydroxy acids.^19,26−28^ These DOX monomers provide a facile route to a range of degradable polyesters such as poly(mandelic acid) upon liberation of small molecules. We speculated that this DOX motif could serve as an entry point into thermosets by expanding to bifunctional derivatives as cross-linkers for a vast array of polymers. In this work, a bifunctional DOX was synthesized and used to prepare a family of polyester networks with properties competitive to those of commercially available non-degradable thermosets. Each network was prepared through a facile one-pot process wherein the ring-opening polymerization of a cyclic ester comonomer occurred simultaneously with cross-linking through the ring-opening of the bifunctional DOX. We demonstrate that the resulting materials have high thermal stability and tunable mechanical properties depending on co-monomer choice. The new resins are both degradable and reprocessable, with both end-of-life fates controlled by the choice of conditions and catalysts, including the ability to recycle glass fiber composites bound by the sustainable resins.

## Results and Discussion

**Scheme 1:**
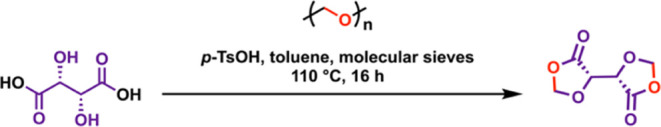
Synthesis of the Bifunctional bisDOX from l-(+)-Tartaric Acid and Paraformaldehyde

The synthesis of monofunctional DOX monomers is well established in the literature.^26,28^ To synthesize the target (4*S*,4′*S*)-[4,4′-bi(1,3-dioxolane)]-5,5′-dione (bisDOX), a modified procedure was followed. The main precursor, l-(+)-tartaric acid, is naturally occurring and often discarded as a by-product of fermentations, ensuring an inexpensive, non-hazardous, and bio-derived starting material.^29^ Previous solvents used in DOX preparation (benzene, cyclohexane, and ethyl acetate) were unproductive. However, toluene proved an optimal solvent for l-(+)-tartaric acid: reflux in toluene with an excess of paraformaldehyde in the presence of *p*-toluenesulfonic acid (*p*-TsOH) as a catalyst, in dilute conditions in order to avoid the formation of oligomeric side products (Scheme 1). Using molecular sieves as drying agents was critical and increased the initial yields from 1.5 to 36%; this improvement was attributed to both their drying ability and slight Lewis acidity, which respectively drove the reaction forward by removing water and enhanced the catalytic activity of the weak *p*-TsOH. The reaction, while low in isolated yield, is surprisingly selective, offering the ability to recycle the unreacted substrate into subsequent reactions. BisDOX can hence be synthesized in a one-step reaction from inexpensive and readily available commercial starting materials isolated through an exceedingly simple purification (see the Supporting Information).

DOX copolymerizations with traditional cyclic esters such as l-lactide and ε-caprolactone systems are well controlled using an aluminum alkoxide catalyst system bearing a salen ligand framework, namely, [Al]OBn (Scheme S1),^28,30,31^ providing a starting point to explore polymerization conditions. Using bisDOX, cross-linked polymers were formed *in situ* through copolymerization with these commercial monomers; previous reports emphasize that the comonomer(s) and the cross-linker must react at similar rates in order for this one-pot process to be efficient.^21,23,32,33^ Following an observed increase in viscosity when heated, the materials were allowed to cure overnight (16 h, 120 °C). Polymerization protocols were optimized by varying the ratio of cross-linker to cyclic ester, while keeping the amount of catalyst constant, and we found that 5 mol % was the optimal cross-linker loading (Table S1). We prepared three different types of resins based on PLA, polycaprolactone (PCL), and poly(δ-valerolactone) (PVL) from the corresponding cyclic esters (l-lactide, ε-caprolactone, and δ-valerolactone), with the latter being the first report of a thermoset exploiting this monomer. We name these resins by reference to the polyester being cross-linked (PLA, PCL, or PVL) and mol % of transesterification catalyst used in network formation (*e.g.*, **PLA-1** represents cross-linked PLA containing 1 mol % [Al]OBn).

The cross-linking progress was monitored by Fourier transform infrared (FTIR) spectroscopy. The distinctive carbonyl stretching frequency corresponding to the bisDOX cross-linker (1785 cm^–1^, Figure S18) disappears over time (Figure S22), suggesting ring opening from the DOX moiety to form the ester. This was corroborated by extrusion of formaldehyde, which following subsequent oligomerization formed a white solid identified as paraformaldehyde. Taken together, these findings suggest full conversion of both cyclic ester and bisDOX rings within the new thermosets. The bisDOX carbonyl stretching frequency is shifted to 1805 cm^–1^ in the reaction mixture, a bathochromic shift that was previously observed in other studies.^24^ For all three resins, the bisDOX carbonyl stretching frequency (1785 cm^–1^, Figure S18) is no longer present in the FTIR spectra after curing, and the only remaining peaks are those of the corresponding polyesters (1749, 1722, and 1725 cm^–1^ for **PLA-1**, **PCL-1**, and **PVL-1**, respectively, Figures S19–S21). This observation suggests full conversion of both cyclic ester and bisDOX rings within the new thermosets.

Conventional linear aliphatic polyesters are highly soluble in organic solvents such as toluene or tetrahydrofuran, whereas cross-linked materials are insoluble and instead swell. Solubility tests on **PLA-1**, **PCL-1**, and **PVL-1** showed none soluble in toluene, hexane, tetrahydrofuran, dimethylformamide, tetrahydrofuran, or water. Quantitative swelling tests performed in dichloromethane revealed high gel contents (*ca.* 80–95%), corroborating efficient cross-linking (Table S2).

This reaction efficiency was maintained when scaled up to the multigram quantities necessary for thermal and mechanical testing. Samples for testing were prepared in one of two ways (see the Supporting Information for more details): (1) directly as tensile bars (*e.g.*, stiffer **PLA-1**) or (2) as uniform thick films that could be cut into bars (*e.g.*, softer **PCL-1** and **PVL-1**). For both preparation methods, the resultant samples were found to have high gel fractions as measured by swelling tests (Table S2). The thermal properties of the materials synthesized were assessed by differential scanning calorimetry (DSC, Figures S3–S13). **PLA-1**, **PCL-1**, and **PVL-1** exhibited slightly increased glass-transition temperatures, *T*_g_, compared to their corresponding homopolymers (Table 1, *T*_g PLA_ = 35–60 °C,^34^*T*_g PCL_ = −60 °C,^35^ and *T*_g PVL_ = −63 °C^36^). This was anticipated since cross-linking reduces the free volume available and hinders chain mobility, thus shifting the *T*_g_ to higher values. A melting transition is observed in the first DSC thermogram for **PLA-1**, but no further crystallization/melting events are observed in the second thermograms (Figure S3). Based on the properties of linear PLLA, a melting transition would be expected throughout repeated DSC analyses, but in this case, cross-linking inhibits reformation of crystalline subdomains. For **PCL-1** and **PVL-1** (Figures S4 and S5), however, a melting transition was still observed, albeit with depressed melting points compared to their linear counterparts. This suggests incomplete inhibition, presumably due to the increased flexibility of the linear chain segments. Above their *T*_g_s, the **PCL-1** and **PVL-1** chains gain sufficient mobility to reorganize into a crystalline structure, mirrored in the cold crystallization events displayed in the DSC thermograms (Figures S4 and S5). All these observations are in line with the visual appearance of our samples, where **PLA-1** is transparent, and **PCL-1** and **PVL-1** are slightly more opaque at room temperature (Figure S2). High-temperature stability is crucial for demanding applications. For all resins, thermal stability was characterized by the 5% mass loss decomposition temperature (*T*_d,5%_, Figures S25–S33 and Table S2). The cross-linked resins exhibit increased thermal stabilities as compared to their homopolymer counterparts.

**Table 1:** Thermal and Mechanical Properties of Thermosets **PLA-1**, **PCL-1**, and **PVL-1**

sample	*T*_g_ (°C)^a^	*T*_m_ (°C)^a^	Δ*H*_m_ (J/g)^a^	*T*_c_ (°C)^b^	Δ*H*_c_ (J/g)^b^	*T*_cc_ (°C)^a^	Δ*H*_cc_ (J/g)^a^	*T*_d,5%_ (°C)^c^	σ_b_ (MPa)^d^	ε_b_ (%)^d^	*E* (MPa)^d^
**PLA-1**	62.7	-	-	-	-	-	-	305	46.7 ± 3.4	4.5 ± 0.4	1268 ± 43
**PCL-1**	–57	24	23.8	–30.1	2.0	–18.0	22.3	308	1.1 ± 0.1	83 ± 11	2.2 ± 0.4
**PVL-1**	–51.3	36.4	40.0	–12.8	29.7	–21.1	1.8	229	5.9 ± 0.3	147 ± 82	67.3 ± 2.6

a Data obtained from the second heating ramp of a DSC experiment.

b Data obtained from the first cooling ramp of a DSC experiment.

c Data obtained from TGA analyses.

d σ_b_ (stress at break), ε_b_ (strain at break), and *E* (Young’s modulus) data obtained from tensile testing measurements of at least five replicates.

**Figure 2:**
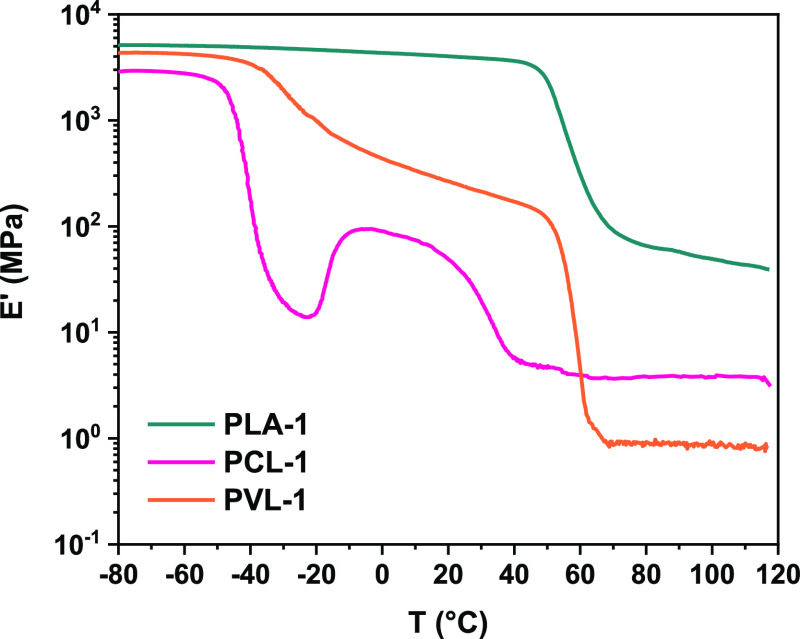
DMTAs of **PLA-1**, **PCL-1**, and **PVL-1**.

Dynamic mechanical thermal analysis (DMTA) experiments were also performed on our cross-linked polyesters. **PLA-1** shows a clear drop in modulus of more than 2 orders of magnitude when heating through *T*_g_, as shown in Figure 2 (teal trace). The plateau modulus appears relatively constant between 80 and 120 °C, consistent with the presence of a network structure. DMTA measurements for **PCL-1** and **PVL-1** (Figure 2, pink and orange traces) instead showed that storage moduli (*E*′) exhibit a similar behavior to that of a semicrystalline polymer.^37^ Heating through *T*_g_ leads to a drop in modulus of approximately 1 order of magnitude. Between *T*_g_ and the melting temperature (*T*_m_), the crystalline regions act as physical cross-links, and thus a relatively high modulus is retained. Heating through *T*_m_ causes a second drop in modulus that is followed by a second plateau originating from the cross-linked network. We propose that the difference in the rubbery moduli between the three networks might be an effect of the relative rate of propagation for each cyclic ester being different to the rate of ring opening of the cross-linker. An additional cold crystallization thermal event influences the DMTA trace of **PCL-1**. The cold crystallization, followed by melting of crystalline domains, manifest as an increase, followed by a decrease in the modulus. If cold crystallization is fast enough relative to the ramp rate of the experiment, then new crystalline domains are forming, leading to an effective increase in modulus. Both thermal events observed by DMTA for **PCL-1** are observed across multiple samples and corroborated by DSC analysis (Figure S4). We are planning further studies to identify the root cause of this unique feature.

**Figure 3:**
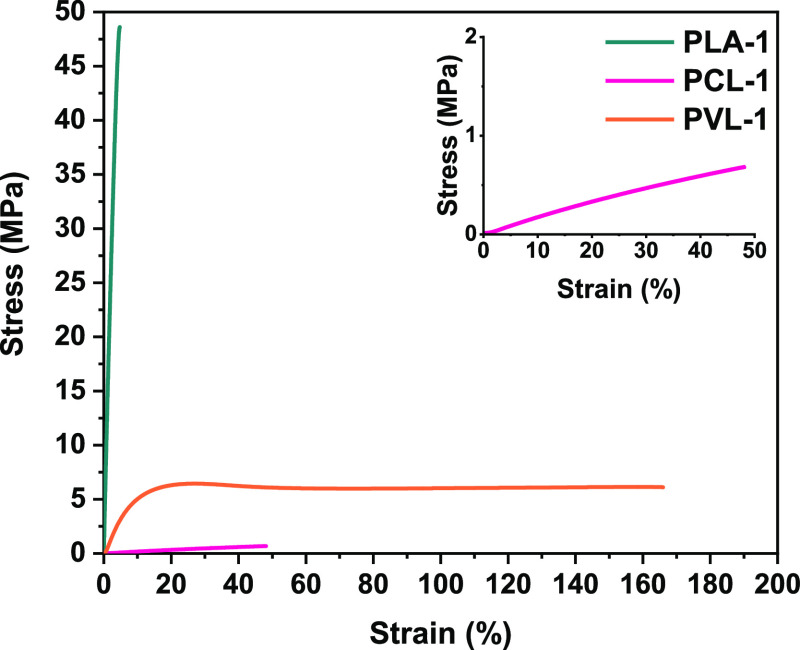
Representative tensile tests of the three polymer networks: **PLA-1** (teal), **PCL-1** (pink), and **PVL-1** (orange).

Tensile testing and shore hardness testing performed at room temperature allowed us to delve deeper into the properties of **PLA-1**, **PCL-1**, and **PVL-1** (Figures 3 and S60 and Tables 1 and S2). **PLA-1** exhibited tensile properties characteristic of a glassy material (Figure 3, teal trace) including a strength at break (σ_b_) of 46.6 ± 3.4 MPa, which is comparable with PLA vitrimers.^25^ The semicrystalline networks **PCL-1** and **PVL-1** are above their *T*_g_ but below their *T*_m_ at room temperature, and thus, we observed elastomeric tensile behavior. They exhibited lower tensile strengths of 1.1 ± 0.1 and 5.9 ± 0.3 MPa but higher elongations at break (ε_b_) of 82.7 ± 11.2 and 147 ± 82%, respectively. The hardness of the three networks was benchmarked relative to that of common materials (Figure S61). A shore D scale test revealed that **PLA-1** (shore hardness 86D) was harder than a 2-part epoxy resin (Araldite, shore hardness 70D) or tough tool handles, such as scissors (shore hardness 74D) or screwdriver (77D, respectively). **PCL-1** and **PVL-1** proved softer, with hardness values of 40D and 48D, respectively. These hardness comparisons once again indicate the influence of the cyclic ester comonomer on the thermomechanical properties of the bisDOX-cross-linked materials.

We next turned our attention to the potential end-of-life pathways of these novel materials. As there was still catalyst embedded in the networks, we postulated that it might facilitate transesterification reactions and thus enable mechanical reprocessing. Indeed, initial tests using **PLA-1**, **PCL-1**, and **PVL-1** showed that we could remold these materials at elevated temperatures (*vide infra*). Therefore, we thought to systematically modify the catalyst content, as this strategy has been shown to tune reprocessing timescales.^25,38^ We have thus prepared PLA, PCL, and PVL networks with increased catalyst loadings (1.5 and 2 mol %) while maintaining a constant cross-linker amount (5 mol %).

In the case of PLA, as the catalyst incorporation was increased past 1 mol %, the network crystallinity was seen to increase, as revealed by DSC analyses (Figures S6 and S9 and Table S2). DMA experiments indicated that the rubbery modulus of **PLA-2** was 1 order of magnitude lower than that for **PLA-1** (Figure S12). This value is directly correlated with the cross-link density (ν_e_), and while **PLA-1** exhibited ν_e_ = 52.9 × 10^–4^ mol cm^–3^, **PLA-2** had ν_e_ 1 order of magnitude lower, which is 9.1 × 10^–4^ mol cm^–3^. The difference in moduli is reflected in the calculated ν_e_ and molar mass between cross-links (*M*_x_) (Table S3) for **PLA-1** and **PLA-2**. We believe that increasing the catalyst and initiator contents led to an increase in the relative rate of cyclic ester ring opening compared to DOX. This would allow for longer segments of PLA to be afforded between cross-links, as evidenced by increasing *M*_x_ values across the series. Crystallization in the slightly longer chains could be more favored in **PLA-1.5** and **PLA-2**, respectively, compared to the highly constrained **PLA-1**. Our hypothesis is that the presence of crystalline segments unfortunately led to **PLA-1.5** and **PLA-2** being extremely brittle.

Tuning catalyst contents for PCL- and PVL-based resins afforded materials that could be cut, allowing for subsequent analyses to be performed. DSC experiments suggested that across the PCL series, there was an increase in the melting enthalpies accompanied by a decrease in the cold crystallization enthalpies (Table S2). This indicates that crystallinity increases across the PCL series as the catalyst content is increased from 1 to 1.5 to 2 mol %. This is strongly correlated with an increase in storage modulus (*E*′) at room temperature, elongation at break σ_b_, strain at break ε_b_, and Young’s moduli across the series (Figures S15 and S35 and Table S2). Conversely, across the PVL family, we observed the opposite trend: a decrease in melting enthalpies accompanied by an increase in the cold crystallization enthalpies, which lead to a decrease in *E*′, σ_b_, ε_b_, and Young’s moduli across the series (Figures S16 and S36 and Table S2).

These experiments highlight the relationship between mechanical properties and crystallinity within our cross-linked networks.

**Figure 4:**
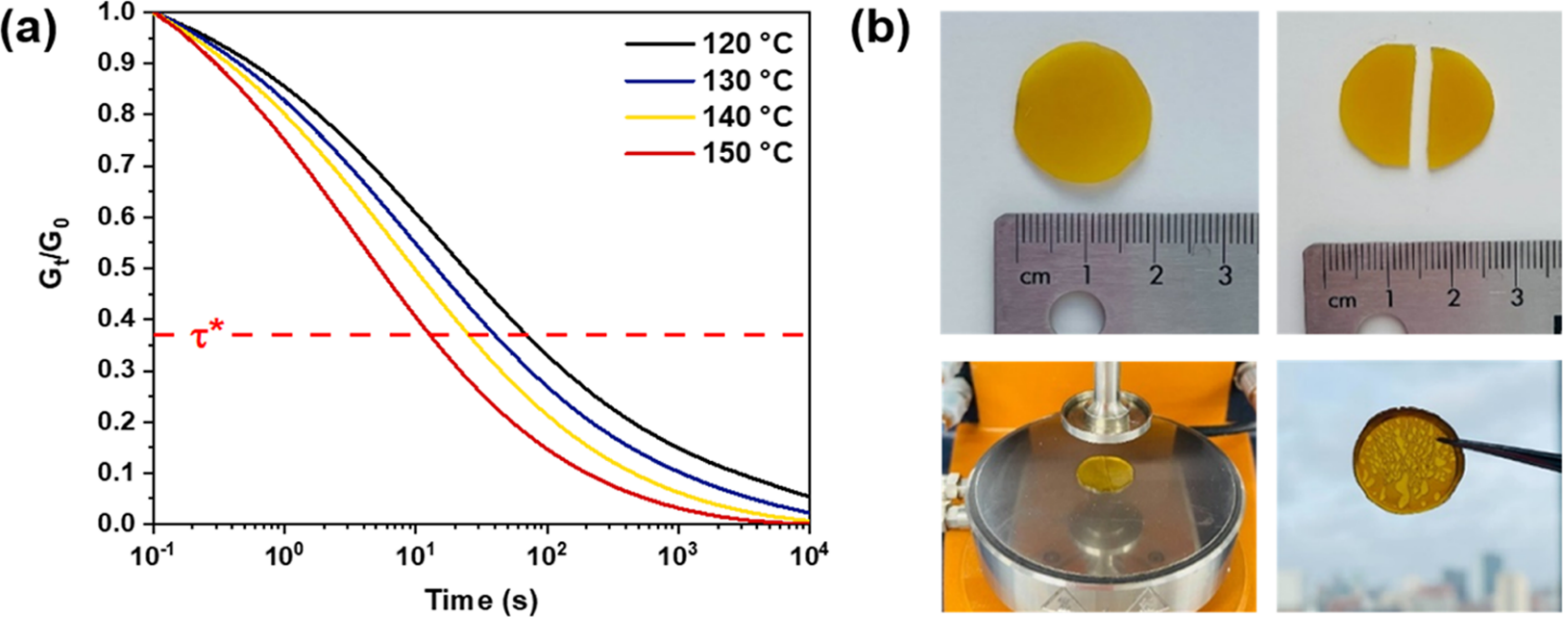
(a) Representative stress relaxation curves of polymer **PVL-2**. (b) **PVL-2** sample cut and then reformed on the rheometer at 150 °C.

To evaluate mechanical reprocessability, stress relaxation analyses (SRAs) are typically used to evaluate dynamic exchanges in the bulk.^39^ We performed SRA experiments on our PCL- and PVL-based resins in the linear viscoelastic regime until the samples had relaxed to 1/*e* (after 1 mean lifetime, τ*) of the initial stress relaxation modulus (Figures 4 and S54–S60). While it is known that thermally activated (*i.e.*, uncatalyzed) transesterification can occur in polyesters, this happens at a much slower rate than when catalyzed.^25,40,41^ From the relaxation behavior of both the PCL and PVL series, it was evident that an increase in catalyst incorporation shortened the relaxation times at a given temperature (Figures S59–S60). For example, Figure 4a shows the SRA of **PVL-2**, and it is clear that the networks are able to relax the stress completely under a reasonable timescale of less than 1 h at 120 °C and remarkably quickly at 150 °C (τ* = 12 s). These SRA results demonstrate that indeed the embedded catalyst enables transesterification. This is particularly exciting since this is, to the best of our knowledge, the first instance of a dynamic cross-linked PVL material. In order to practically demonstrate the reprocessability enabled by the dynamic nature of the **PVL-2** network, we cut it into two pieces and then reformed it back into a homogeneous sample on the rheometer at 150 °C (Figure 4b). The reprocessed samples showed identical thermal properties as the original samples (Figures S11 and S13) with only a slight suppression of the cold crystallization. The FTIR spectrum was also unchanged after reprocessing (Figure S24). Moreover, the characteristic relaxation times (τ*) for **PVL-2** at various temperatures were fitted to an Arrhenius model (Figure S54) from which the activation energy of stress relaxation *E*_a_ was extracted to be 33 kJ mol^–1^. Since this is the first report of a post-curing [Al] complex catalyzing a transesterification process, we are only able to state that the activation energy is lower than Brønsted acid-catalyzed exchanges (*ca.* 55 kJ mol^–1^),^42^ and lower than the literature value of *E*_a_ for Sn(Oct)_2_-catalyzed transesterification in PLA melt, which was reported as 83 kJ mol^–1^.^25,43^ We found that our **PCL-2** could also be reprocessed at 150 °C using a hot press. Similar to **PVL-2**, the thermal properties and characteristic FTIR frequencies remained unchanged (Figures S10, S12, and S23). Such results are promising, but further investigation into the robustness and repeatability of this catalyzed exchange is needed. This is particularly important when considering the air-sensitive nature of the catalyst whose integrity is necessary for efficient transesterification reactions.

**Figure 5:**
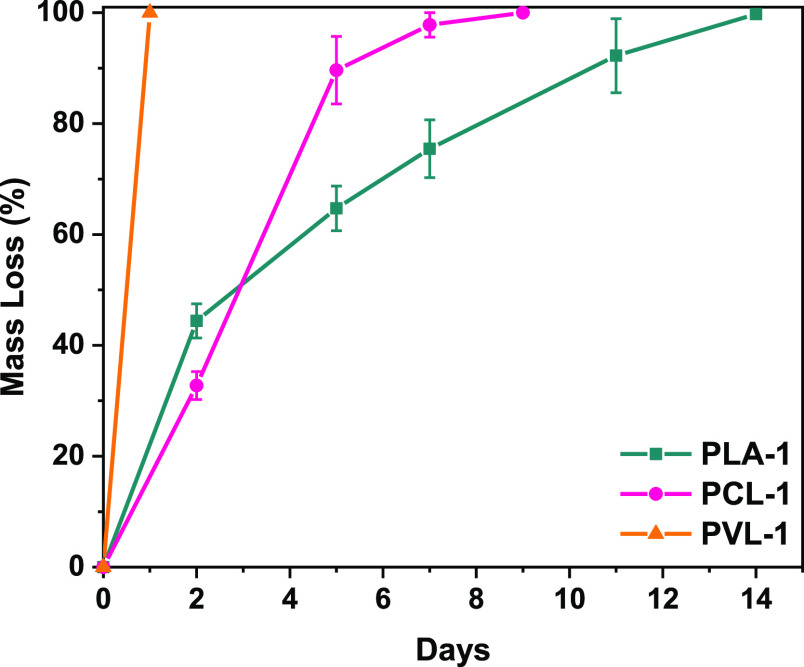
Degradation profiles for **PLA-1**, **PCL-1**, and **PVL-1** samples under basic conditions (1 M NaOH_aq_).

Although traditional thermosets are notoriously non-degradable,^2,9−11,15,21,23,44^ the materials presented herein have labile ester bonds that we envisioned would unlock degradation as a potential end-of-life option.^45^ We first investigated whether the resins could be degraded under accelerated hydrolysis conditions. We were concerned that the network stiffness and hydrophobicity would not allow solvent penetration, hence inhibiting degradation,^21,46^ but a variety of preliminary experiments suggested otherwise. Thin, rectangular pieces (40 × 4 × 1 mm) of **PLA-1**, **PCL-1**, and **PVL-1** were immersed in phosphate-buffered saline (PBS 1 M) and 1 M aqueous NaOH at ambient temperatures. Moreover, in order to allude to their suitability for applications in the built environment, we investigated their hydrolytic degradation in aqueous acidic media and in artificial seawater. We detected virtually no mass loss for the samples in PBS or artificial seawater. Furthermore, in acidic conditions, **PLA-1** and **PCL-1** displayed no mass loss, whereas **PVL-1** lost 27% mass over 5 weeks. However, in the presence of NaOH, we were able to completely degrade **PLA-1** in 14 days, **PCL-1** in 10 days, and **PVL-1** in less than 1 day (Figure 5). These findings are in line with previous reports that demonstrate that PCL degrades slower than PLA.^47^ When investigating the effect of surface area over degradation kinetics, we found that **PLA-1** bars (60 × 12 × 3 mm) degrade in 35 days, more than double the time it takes for **PLA-1** films (40 × 4 × 1 mm) to degrade (Figure S47). Enabling the mild, chemically triggered degradation is an exciting avenue to pursue, but identifying the degradation products informs whether such degradation could be leveraged for chemical recycling. Qualitative ^1^H NMR analyses (Figures S37–S40) as well as GPC measurements (Figures S48–S50 and Table S4) suggested that our cross-linked materials degraded to short-chain, soluble oligomers as well as tartaric acid. A promising avenue is opened for these materials to be circularized since we are able to recover l-(+)-tartaric acid, the main feedstock for synthesizing bisDOX (Figure S37). This platform could allow for isolation of l-(+)-tartaric acid, along with oligomer separation. PLA oligomers could theoretically allow for depolymerization to l-lactide, whereas the other oligomers obtained as degradation products could be employed to prepare thermosets with a similar structure and performance, which is central to a sustainable development of cross-linked polymers.^48−50^

Degradation in aqueous environments proved successful on a reasonable timescale (1–14 days) depending on the surface area, but we hypothesized that we could enhance the degradation rates by introducing a stronger base, namely, 1,8-diazabicyclo[5.4.0]undec-7-ene (DBU), well known for aiding the depolymerization of PET.^51^ To test this, we prepared a 1 M solution of DBU in acetonitrile, a polar solvent that swells the networks, and then added thin, rectangular pieces (40 × 4 × 1 mm) of **PLA-1**, **PCL-1**, and **PVL-1**. Remarkably, complete dissolution of all three networks was observed in approximately 30 min under ambient conditions. ^1^H NMR spectroscopy of the degraded resins (Figures S41–S46), along with GPC analyses (Figures S51–S53), revealed that the products correspond once again to short-chain PLA, PCL, and PVL oligomers (Table S3).

**Figure 6:**
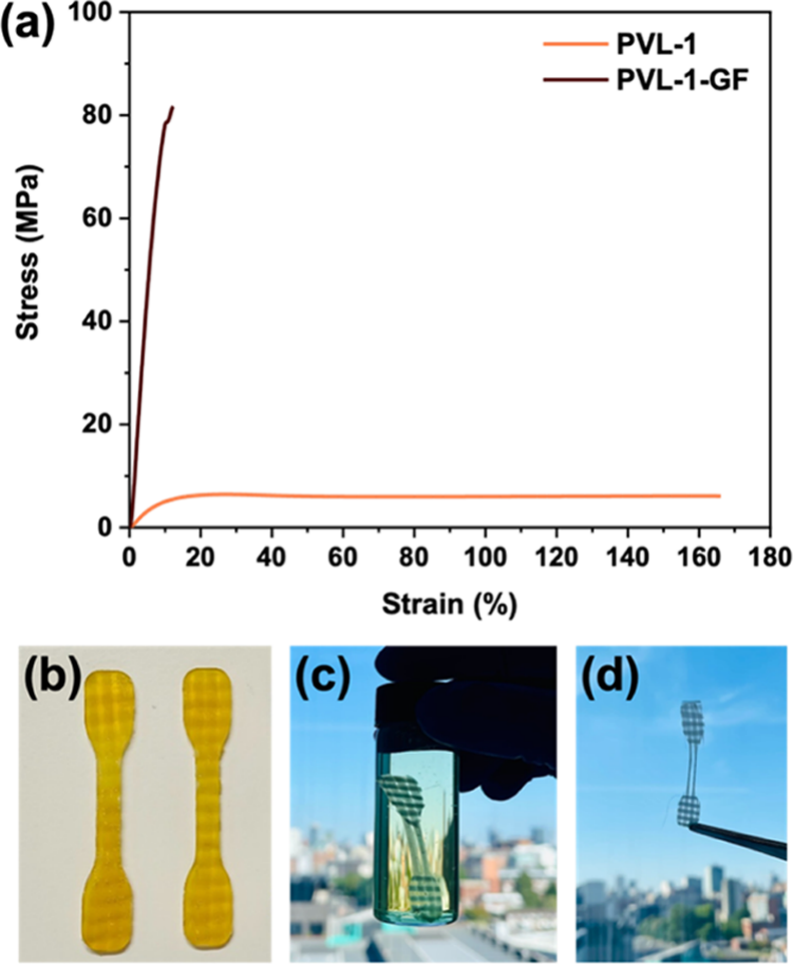
(a) Representative tensile testing for the composite **PVL-1-GF** contrasted to **PVL-1**, its thermoset counterpart. (b) **PVL-1-GF** dumbbells. (c) **PVL-1-GF** in 1 M aqueous NaOH. (d) Pristine glass fiber recovered after degradation of the composite matrix.

Traditional thermosets (*e.g.*, epoxy resins) are typically used for structurally demanding applications calling for superior structural properties.^11,52−54^ We were astounded by the promising material properties shown, along with their potential for degradability, so we have thus aimed to expand the scope of our cross-linked polyesters to fiber-reinforced composites (FRCs). As a proof of concept, the **PVL-1** formulation was used to impregnate one layer of plain-woven glass fiber of 290 gsm. After curing for 16 h at 120 °C, this resulted in a yellow transparent composite (**PVL-1-GF**). Reinforcing our original **PVL-1** resin led to an enhancement in tensile properties, allowing for a Young’s modulus of 0.975 GPa and a tensile strength of 73.8 MPa (Figure 6a). More importantly, the polymer matrix can be readily degraded (Figure 6c) to afford recovery of the pristine glass fiber (Figure 6d). This is a promising outcome, suggesting that the scope of degradable cross-linked polyesters can be expanded to include FRCs without compromising the cross-linking efficiency. Further work is needed to improve the thermal properties and tune the activation energy for flow to target engineering applications. The full exploration of these composites is part of an upcoming study.

## Conclusions

The synthesis of a family of reprocessable and degradable cross-linked polyesters using a novel bis(1,3-dioxolan-4-one) molecule and traditional cyclic esters such as l-lactide, ε-caprolactone, and δ-valerolactone unlocks high-performance, sustainable resins. The thermal and mechanical properties of these cross-linked polyesters could be tuned by the cyclic ester component and the catalyst content, accessing properties ranging from rigid materials to elastomeric solids. The strongest of these thermosets are built with PLA, with hardness competitive with that of conventional thermosets. Degradation is facile under accelerated hydrolysis conditions, with complete mass loss in under 2 weeks. Using an organocatalyst, DBU, further accelerated degradation rates such that the materials were completely solubilized in less than 30 min, highlighting the potential for chemical recycling. Moreover, the vitrimeric nature of the resins also allows for mechanical reprocessing at increased temperatures. This is, to the best of our knowledge, the first report of reprocessable networks containing aluminum salen as a catalyst, along with the first report of a dynamic PVL material. To showcase the potential application of these new resins, we have used glass fibers as reinforcement for our cross-linked PVL. The degradable matrix allowed for the pristine glass fibers to be recovered following base-catalyzed hydrolysis. The materials synthesized thus prove to be promising candidates as renewable and degradable alternatives to conventional thermosets.

## Supporting Information Available

Materials, methods, additional raw data, and further experimental details (PDF)
