# Supplementary material for: Degradable and Reprocessable Resins from a Dioxolanone Cross-Linker
Source: Macromolecules. 2023 Feb 9;56(4):1625–32. doi: 10.1021/acs.macromol.2c02560 (PMC9979638; doi:10.1021/acs.macromol.2c02560)
Supplement: Supplementary file 1 — ma2c02560_si_001.pdf [file ma2c02560_si_001.pdf]

Supporting Information **for**

Degradable and Reprocessable Resins From a Dioxolanone Cross-linker

Theona Șucu[a,b], Meng Wang[a,b] and Michael P. Shaver* [a,b]

[a] Department of Materials, Engineering Building A, The University of Manchester, Oxford Road, M13 9PL, United Kingdom;

[b] Sustainable Materials Innovation Hub, Henry Royce Institute, The University of Manchester, Manchester, M13 9PL, United Kingdom.

*michael.shaver@manchester.ac.uk

Pages - 41

Figures - 61

Tables - 4

Schemes - 1

General Considerations. All oxygen and moisture-sensitive reactions were performed under an inert atmosphere using standard Schlenk techniques on a dual-manifold Schlenk line equipped with an in-line gas drying column containing a copper catalyst or in a nitrogen-filled glovebox, unless otherwise stated. All glassware and moulds were pre-dried in an oven at 200 °C overnight.

Materials. All reagents were used as received unless otherwise indicated. Anhydrous toluene was obtained from an MBraun 7 Solvent Purification System containing alumina and copper catalysts and degassed *via* three successive freeze-pump-thaw cycles prior to use. $CDCl_3$ (99.8 atom% D, Aldrich), ε-caprolactone (98%, Sigma-Aldrich), δ-valerolactone (>95 %, Aldrich), benzyl alcohol (Fluorochem Ltd.) were all stirred over CaH_2 (Aldrich) and distilled under reduced pressure. L-lactide (99%, Corbion) was stored in the vacuum oven at 25 °C overnight, followed by a double sublimation under reduced pressures and stored under inert atmosphere. L-(+)-tartaric acid (Aldrich), 2,2-dimethyl-1,3-propanediamine (Fluorochem Ltd.), trimethyl aluminium (2.0 M in hexanes, Aldrich), *p*-toluenesulfonic acid monohydrate (Aldrich), paraformaldehyde (Aldrich) were all used as received.

Synthesis of bisDOX ((4*S*,4'*S*)-[4,4'-bi(1,3-dioxolane)]-5,5'-dione) cross-linker. L-(+)-tartaric acid (0.33 mol, 50 g), paraformaldehyde (1.33 mol, 40 g) and *p*-TsOH·H_2O (0.065 mol, 11.53 g) were dissolved in toluene (700 mL). Previously dried molecular sieves (4 Å) were added and the reaction mixture was refluxed at 110 °C overnight. The mixture was allowed to cool to room temperature and ethyl acetate (150 mL) was added to the reaction mixture. It was then washed with saturated $NaHCO_3$ solution (250 mL), deionised water (250 mL) and saturated aqueous NaCl solution (250 mL). The organic fraction was dried over $MgSO_4$ and concentrated *in vacuo*. It was then dissolved in THF and precipitated from hexane. The product was afforded as white fluffy crystals in 36 % isolated yield.
^{1}H NMR ($CDCl_3$, 400 MHz): δ 5.60 (s, 2H), 5.55 (s, 2H), 4.70 (s, 2H);
^{13}C NMR ($CDCl_3$, 126 MHz): δ 170.08, 95.97, 72.61.
HR-ESI-MS calculated for $C_6H_6O_6$ $[M+H]^+$ 175.0237, found 175.0231.

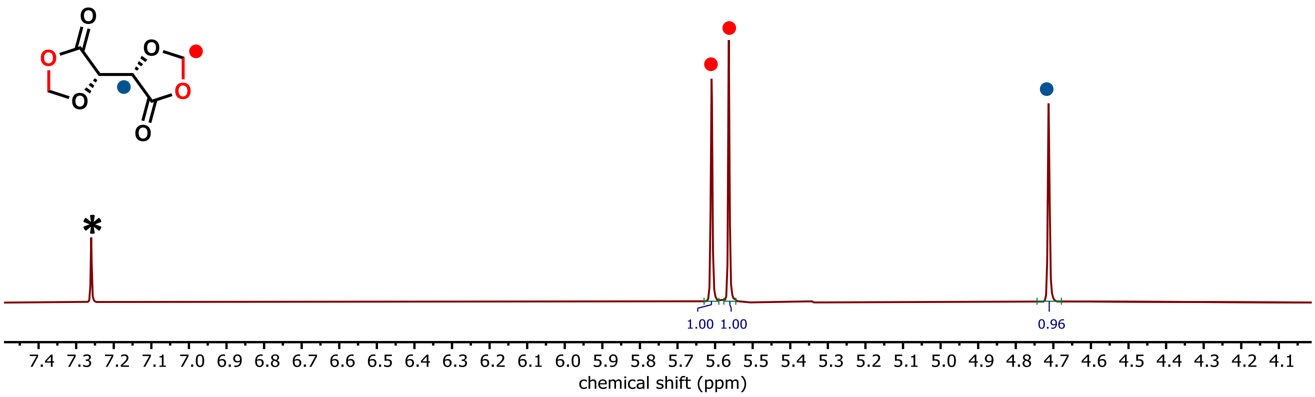

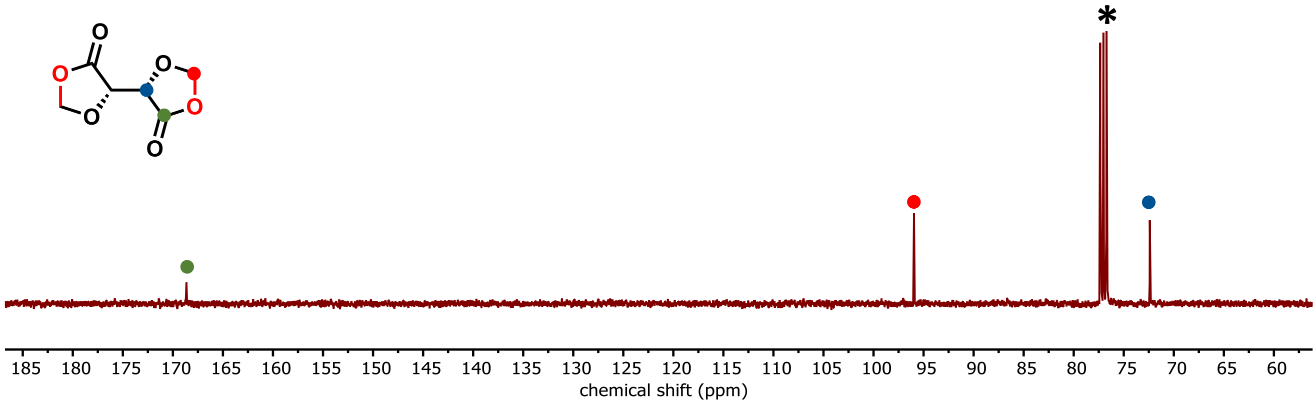

Figure S1. ^{1}H NMR spectrum ($CDCl_3$, 400 MHz) (top) and ^{13}C NMR spectrum ($CDCl_3$, 126 MHz) (bottom) of bisDOX.

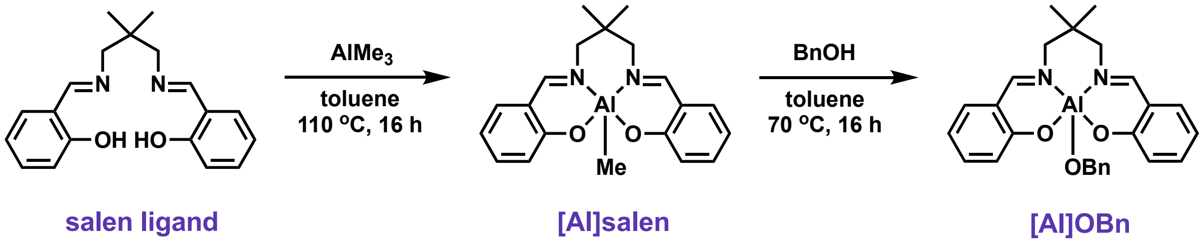

Scheme S1. Synthesis of the pre-initiated catalyst [Al]OBn.

Synthesis of salen ligand (N,N'-bis(salicylidene)-2,2-dimethyl-1,3-propanediamine). The salen ligand was prepared based on a literature procedure.[1] A solution of 2,2-dimethyl-1,3-propanediamine (97.8 mmol, 10 g) in ethanol was added to a rapidly stirring solution of salicylaldehyde (195.7 mmol, 23.11 g) in ethanol at room temperature. The reaction was then warmed to reflux and stirred for 3 h.

On cooling, the desired product precipitated as yellow crystals and after washing with cold ethanol, isolating by filtration and drying *in vacuo,* a recovered yield of 78% was recorded.
^{1}H NMR ($CDCl_3$, 500 MHz)*:* δ 8.34 (s, 2H), 7.36-7.24 (m, 2H), 6.99-6.96 (m, 2H), 6.92-6.86 (m, 2H), 3.49 (s, 4H), 1.08 (s, 6H).

Synthesis of aluminium salen pre-catalyst, [Al]salen. The catalyst was prepared based on a literature procedure.[1] In a nitrogen-filled glovebox, to a vigorously stirred solution of *N,N'*-bis(salicylidene)-2,2-dimethyl-1,3-propanediamine (6.45 mmol, 2 g) in toluene (30 ml) was added $AlMe_3$ (3.3 mL of a 2.0 M solution in toluene, 6.45 mmol) dropwise. The Schlenk flask was sealed and removed from the glovebox. The reaction mixture was stirred at 110 °C overnight and after concentration *in vacuo*, pale-yellow crystals formed in 64% yield. They were washed with dry toluene and dried under vacuum.
^{1}H NMR ($CDCl_3$, 500 MHz)*:* δ 8.07 (s, 2H), 7.40-7.33 (m, 2H), 7.18 (m, 2H), 7.01 (m, 2H), 6.71(m, 2H), 3.84 (d, *J* = 12.1 Hz, 2H), 3.24 (d, *J* = 12.1 Hz, 2H), 1.15 (s, 3H), 0.98 (s, 3H), -0.81 (s, 3H).

Synthesis of aluminium salen pre-initiated catalyst, [Al]OBn. In a nitrogen-filled glovebox, to a vigorously stirred solution of precatalyst (4.22 mmol, 1.48 g) in toluene, benzyl alcohol (4.65 mmol, 0.5028 g) was added dropwise. The Schlenk flask was sealed and removed from the glovebox. The reaction mixture was stirred at 70 °C overnight and white crystals formed in 79% yield.
^{1}H NMR ($CDCl_3$, 500 MHz)*:* δ 8.08 (s, 2H), 7.41 (m, 2H), 7.22-7.03 (m, 10H), 6.78-6.72 (m, 2H), 4.70 (s, 2H), 3.71 (d, 2H), 3.23 (d, 2H), 0.95 (s, 3H), 0.93 (s, 3H).

Representative cross-linking procedure for PLA. In a nitrogen filled glovebox, the catalyst (0.299 mmol, 0.1327 g for **PLA-1**), cross-linker (1.499 mmol, 0.2611 g) and monomer (0.03 mol, 4.3334 g) were weighted out directly in an oven-dried vial. Due to the powder nature of the contents, they had to be mixed thoroughly using a spatula. The vial was then sealed, removed from the glovebox and placed in a glovebag (Aldrich, AtmosBag). After degassing the contents three times, the mixture was placed in pre-heated steel moulds pre-sprayed with a PTFE mould-release spray (Rocol). The mixture was further mixed using a spatula, until the contents were melted. The moulds were covered, and the samples were left to cure at 120 °C. After 16 h, the samples were cooled down, removed from the moulds and subsequently used.

Representative cross-linking procedure for PCL and PVL. In a nitrogen filled glovebox, the catalyst (0.8761 mmol, 0.3874 g for **PCL-1**; 0.9988 mmol, 0.4416 g for **PVL-1**), cross-linker (4.99 mmol, 0.8688 g for PCL; 4.38 mmol 0.7627 g for PVL) and monomer (0.088 mol ε-caprolactone,

0.099 mol δ-valerolactone, 10 g) were weighted out directly in an oven-dried vial. The vial was then sealed, removed from the glovebox and the contents were ultrasonicated for 5 minutes to ensure good mixing. They were then and placed in a glovebag (Aldrich, AtmosBag). After degassing the contents, the mixture was poured in a pre-heated Petri dish (borosilicate, 94 mm diameter) pre-sprayed with a PTFE mould-release spray (Rocol). The samples were covered with a lid (borosilicate, 100 mm diameter) and were left to cure at 120 °C. After 16 h, the samples were cooled down and peeled from the dishes.

Polyester resins hydrolysis experiments. Thin film samples were cut from the polyester resins, and they were weighted and placed in a 28 mL scintillation vial and immersed in the solutions of interest. The vials were then placed in an orbital shaker and the temperature was set at 25 °C. At certain time intervals, the polyester samples were removed, padded dry and weighted. The data points reported have been averaged over 3 samples.

Representative composite synthesis procedure. A standard **PVL-1** formulation was prepared as above and the liquid mixture was poured on a pre-cut glass fibre mat (290g Plain Weave Woven Glass Cloth) previously dried in a vacuum oven (60 °C, 6 h). Samples were peeled out of the Petri dish and cut for tensile testing using an ISO-37-3 die cutter.

Polyester resins reprocessing experiments. The polyester resins samples were cut in small pieces and placed in a hot press at 150 °C for 1 h.

Swelling tests. Swelling tests were performed using dichloromethane. A small amount of each cross-linked sample was immersed in dichloromethane for 24 h before the solvent was decanted. The swollen sample was then dried under vacuum for 24 h or until constant weight before the final mass was measured. The gel fraction was calculated by taking the ratio of the final mass to the initial mass, with values given as averages of triplicate measurements.

Characterization. All **^{1}H and ^{13}C NMR** spectra were obtained on Bruker Avance 400 and 500 MHz instruments. All spectra were obtained at ambient temperatures unless otherwise stated. The chemical shifts (δ) and coupling constants (J) were recorded in parts per million (ppm) and Hertz (Hz) respectively. The residual solvent peaks of the deuterated solvents were used as reference and spectra was recorded relative to it.

Differential scanning calorimetry (DSC) was performed on a DSC 2500 TA instrument using heat (–80 to 220 °C)/cool (220 to –80 °C)/heat (–80 to 220 °C) cycles at a rate of 10 °C min^{-1}. Values of T_g and T_m were obtained from the second heating scan. All analyses were performed in triplicate.

Thermogravimetric analysis (TGA) was performed using a TA Instruments Q800 instrument. The samples (10-25 mg) were heated in nitrogen from room temperature to 600 °C at a rate of 5 °C min^{-1}.

Fourier transform infrared (FTIR) spectra were obtained using a Bruker INVENIO instrument with a Smart Orbit diamond single bounce crystal. The data reported are the average of 32 scans and were acquired using a resolution of 4 cm^{-1} at room temperature.

Stress relaxation experiments were performed on a stress-controlled TA DH2 rheometer with a 20 mm parallel plate geometry. Temperature (90-150 °C) was controlled by a Peltier plate. The samples were allowed to equilibrate at the desired temperature for 15 minutes, after which they were subjected to an instantaneous strain of 2 %. The stress decay was monitored, until the stress relaxation modulus had relaxed to at least 37 % (1/e) of its initial value. This was performed in triplicate for each sample.

Dynamic mechanical thermal analysis (DMTA) was performed using a Q800 DMA (TA Instruments, USA) operating in either a three point bend mode on rectangular PLA bar samples (60 mm × 12 mm × 3 mm) or in a thin film mode on rectangular PCL/PVL samples (7 mm × 5 mm × 1.5 mm) with a heating regime of −80 °C to 120 °C at 3 °C min^{-1}, and a measurement frequency of 1 Hz with an oscillating strain of 0.05%. All analyses were performed in triplicate.

The cross-linking density was calculated following a literature procedure and using the storage modulus (E') at 100 °C and equation 1, where E' and G' are the storage and shear moduli, respectively, R is the universal gas constant, T is the absolute temperature in the rubbery region (*ca.* 373 K).[2]

$$E'(T) = 3G'(T) = 3RT\nu_e = \frac{\rho RT}{M_x} \qquad (1)$$

Tensile tests were conducted on a Static Testing Instron 3344L3928 fitted with either a 100 N or 500 N load cell. Elastomeric samples, matching ISO 37-3, were tested at constant speeds of 200 mm/min. Rigid samples, matching ISO 527-2-1BB, were tested at constant speeds of 0.5 mm/min. The strain at break (ε_b), stress at break (σ_b), and Young's modulus (E) were measured and calculated. A minimum of 5 samples were tested per sample batch accordant with both ISO standards used. Samples according to the respective ISO standards for tensile testing were obtained either by CNC milling performed in the University of Manchester workshops (for PLA samples), or by using an ISO-37-3 die cutter mounted on a 8 kN toggle press (for PCL and PVL).

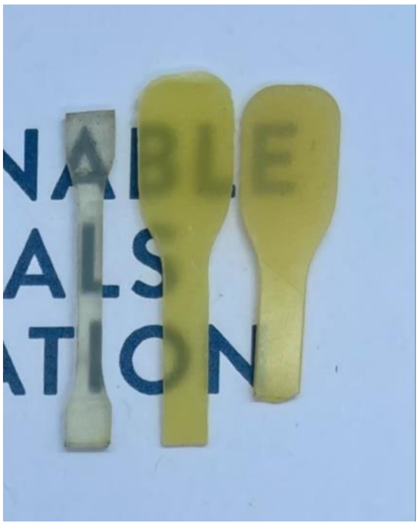

Figure S2. Picture of the obtained thermosets as (broken) dumbells: transparent **PLA-1** CNC milled to ISO 527-2-1BB (left), the more opaque **PCL-1** (middle) and **PVL-1** (right) as broken ISO 37-3 specimens after tensile testing.

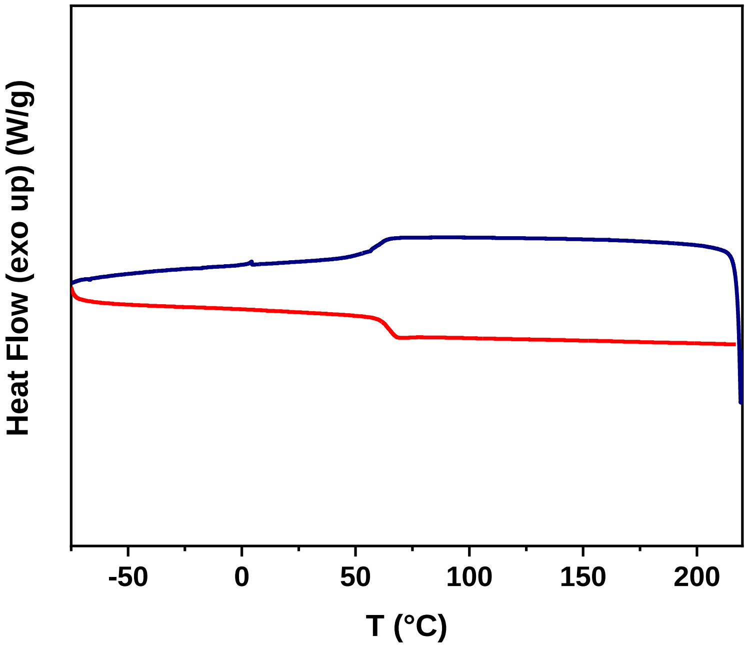

Figure S3. DSC trace of **PLA-1**, with first cooling (blue) and second heating (red).

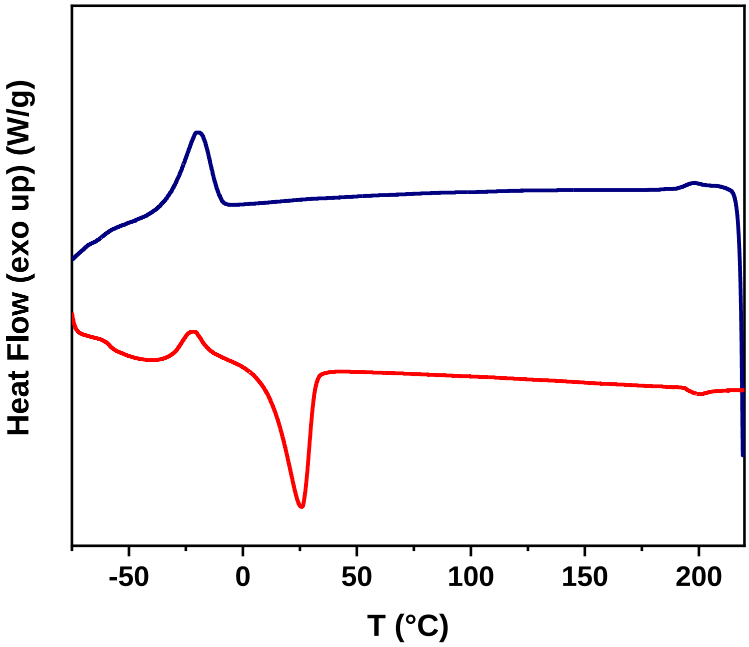

Figure S4. DSC trace of **PCL-1**, with first cooling (blue) and second heating (red).

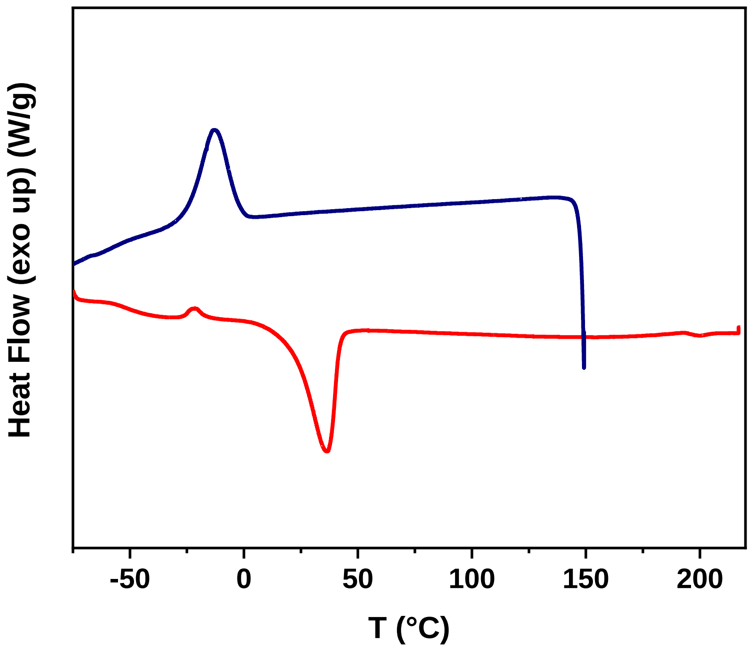

Figure S5. DSC trace of **PVL-1**, with first cooling (blue) and second heating (red).

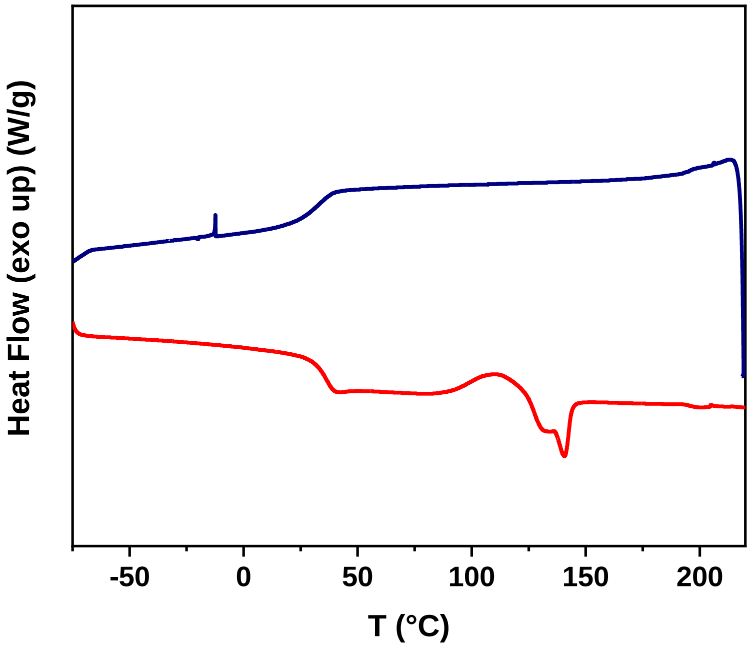

Figure S6. DSC trace of **PLA-1.5**, with first cooling (blue) and second heating (red).

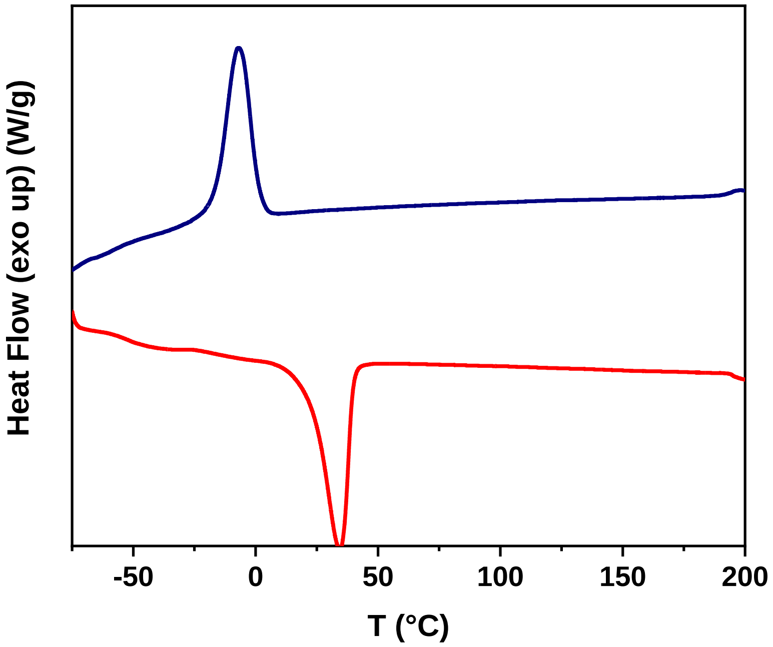

Figure S7. DSC trace of **PCL-1.5**, with first cooling (blue) and second heating (red).

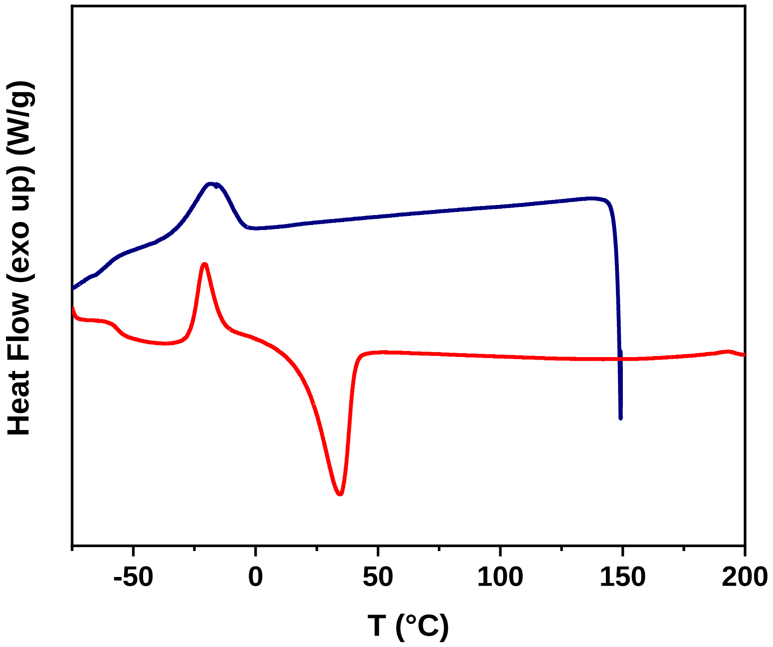

Figure S8. DSC trace of **PVL-1.5**, with first cooling (blue) and second heating (red).

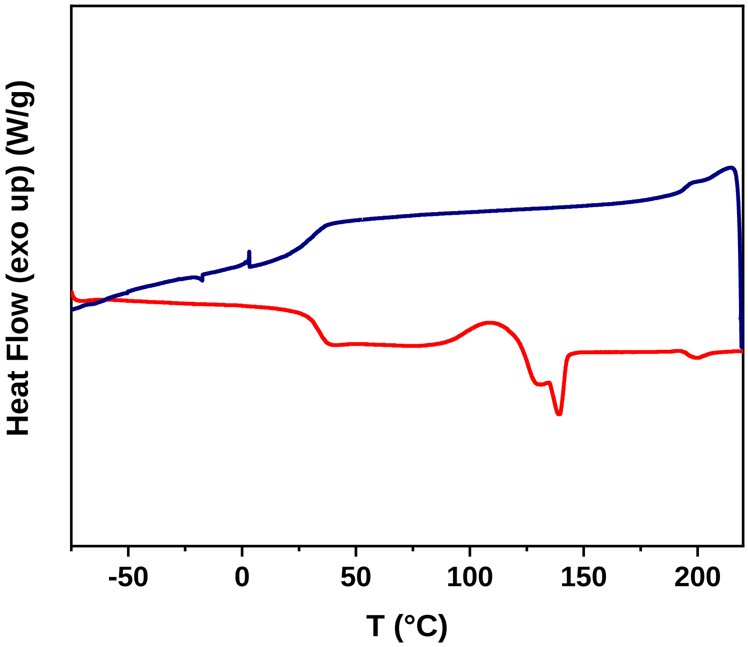

Figure S9. DSC trace of **PLA-2**, with first cooling (blue) and second heating (red).

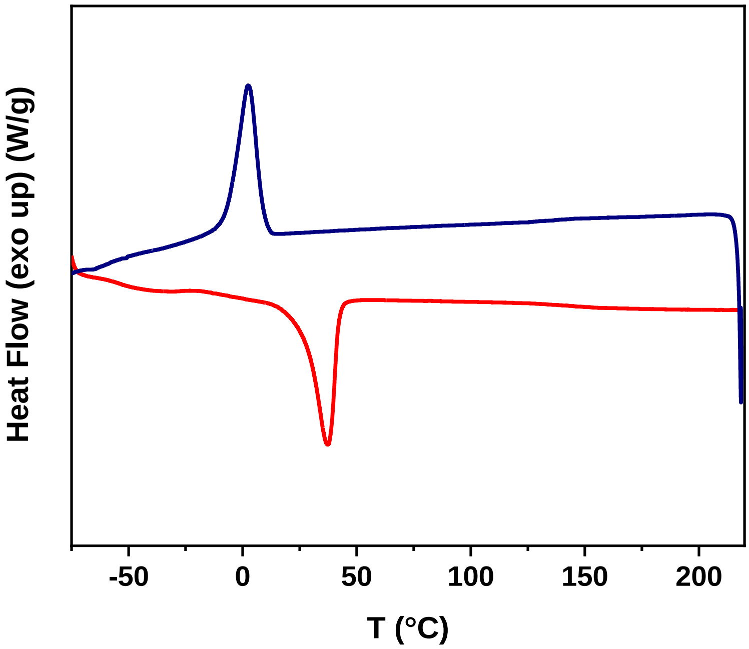

Figure S10. DSC trace of **PCL-2**, with first cooling (blue) and second heating (red).

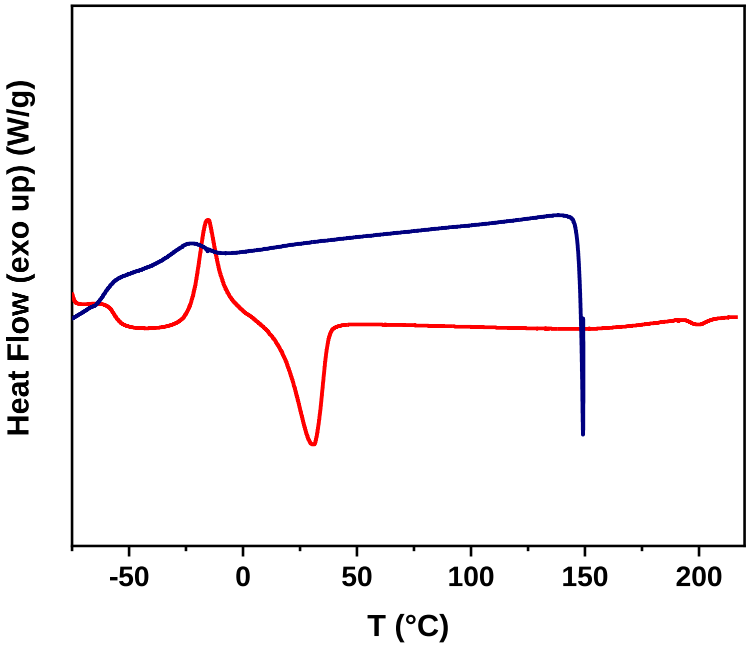

Figure S11. DSC trace of **PVL-2**, with first cooling (blue) and second heating (red).

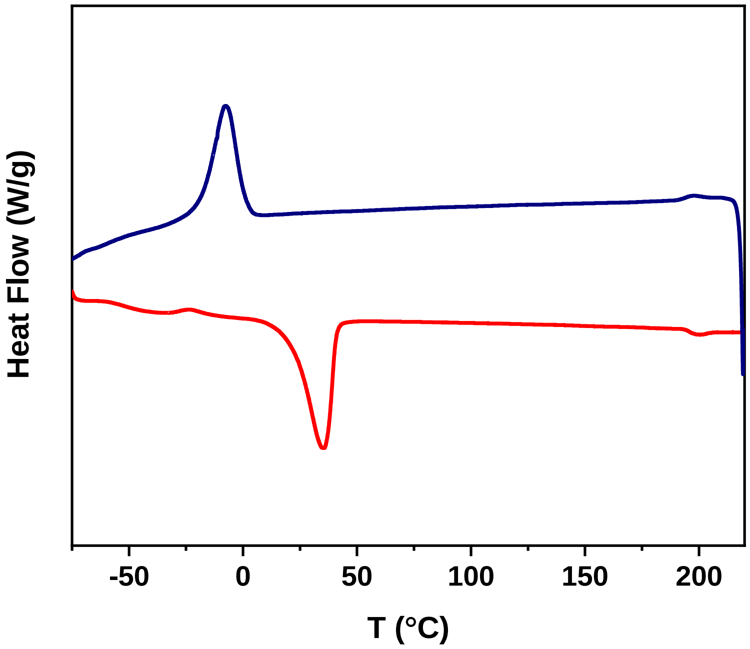

Figure S12. DSC trace of reprocessed **PCL-2**, with first cooling (blue) and second heating (red).

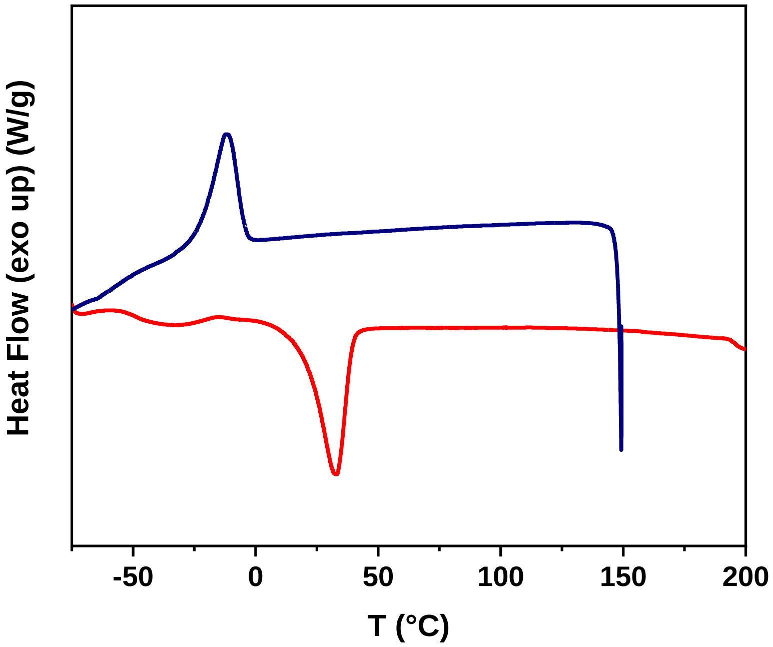

Figure S13. DSC trace of reprocessed **PVL-2**, with first cooling (blue) and second heating (red).

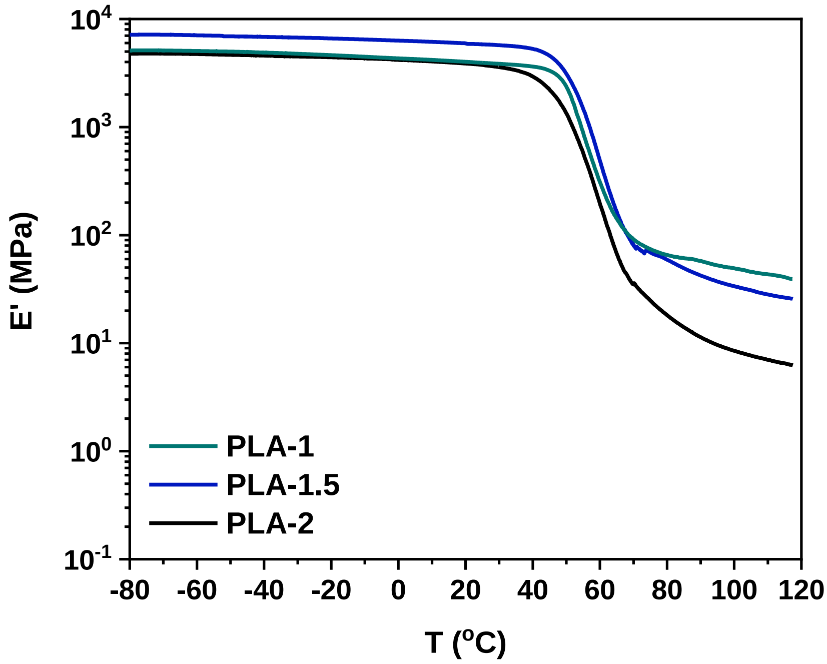

Figure S14. Dynamic mechanical thermal analyses (DMTA) of **PLA-1**, **PLA-1.5** and **PLA-2**, showing the storage modulus as a function of temperature.

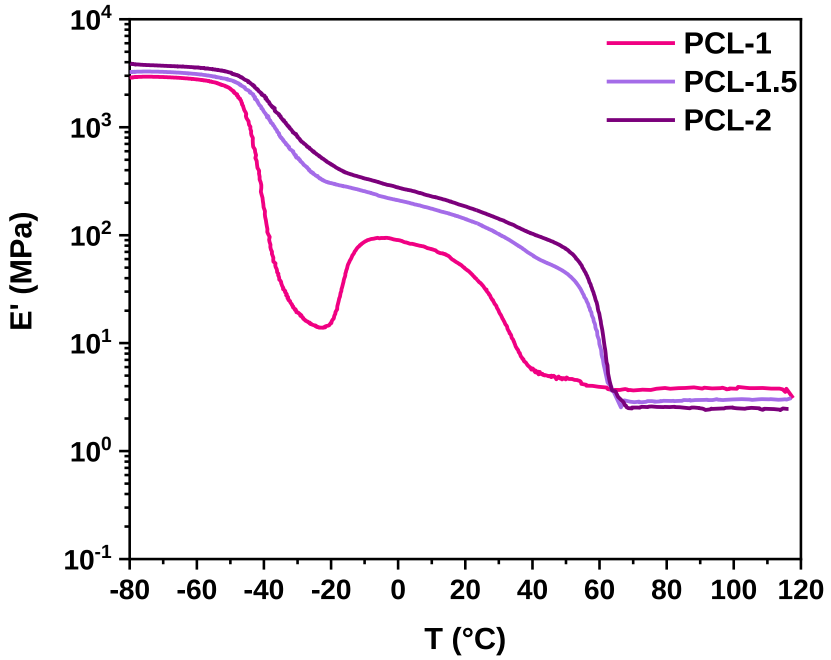

Figure S15. Dynamic mechanical thermal analyses (DMTA) of **PCL-1**, **PCL-1.5** and **PCL-2**, showing the storage modulus as a function of temperature.

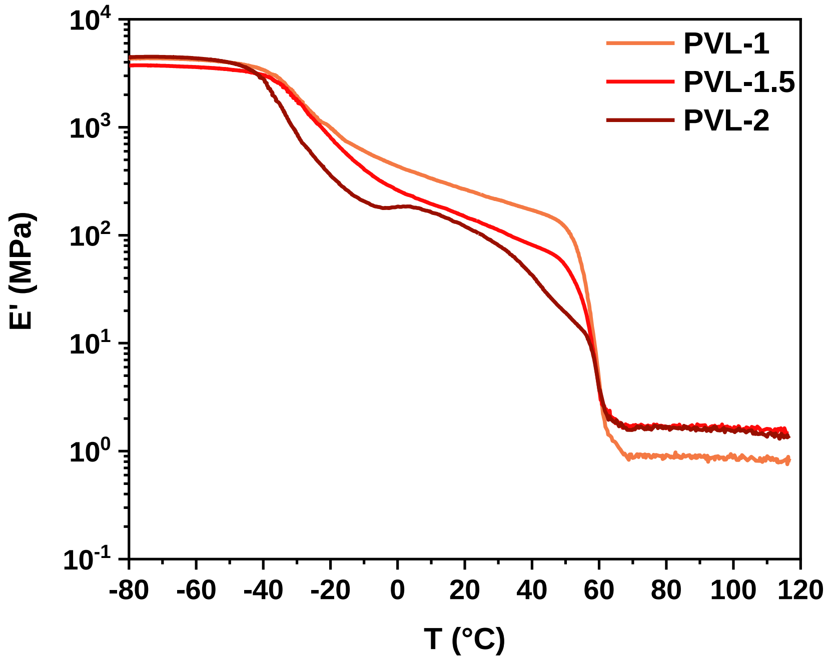

Figure S16. Dynamic mechanical thermal analyses (DMTA) of **PVL-1**, **PVL-1.5** and **PVL-2**, showing the storage modulus as a function of temperature.

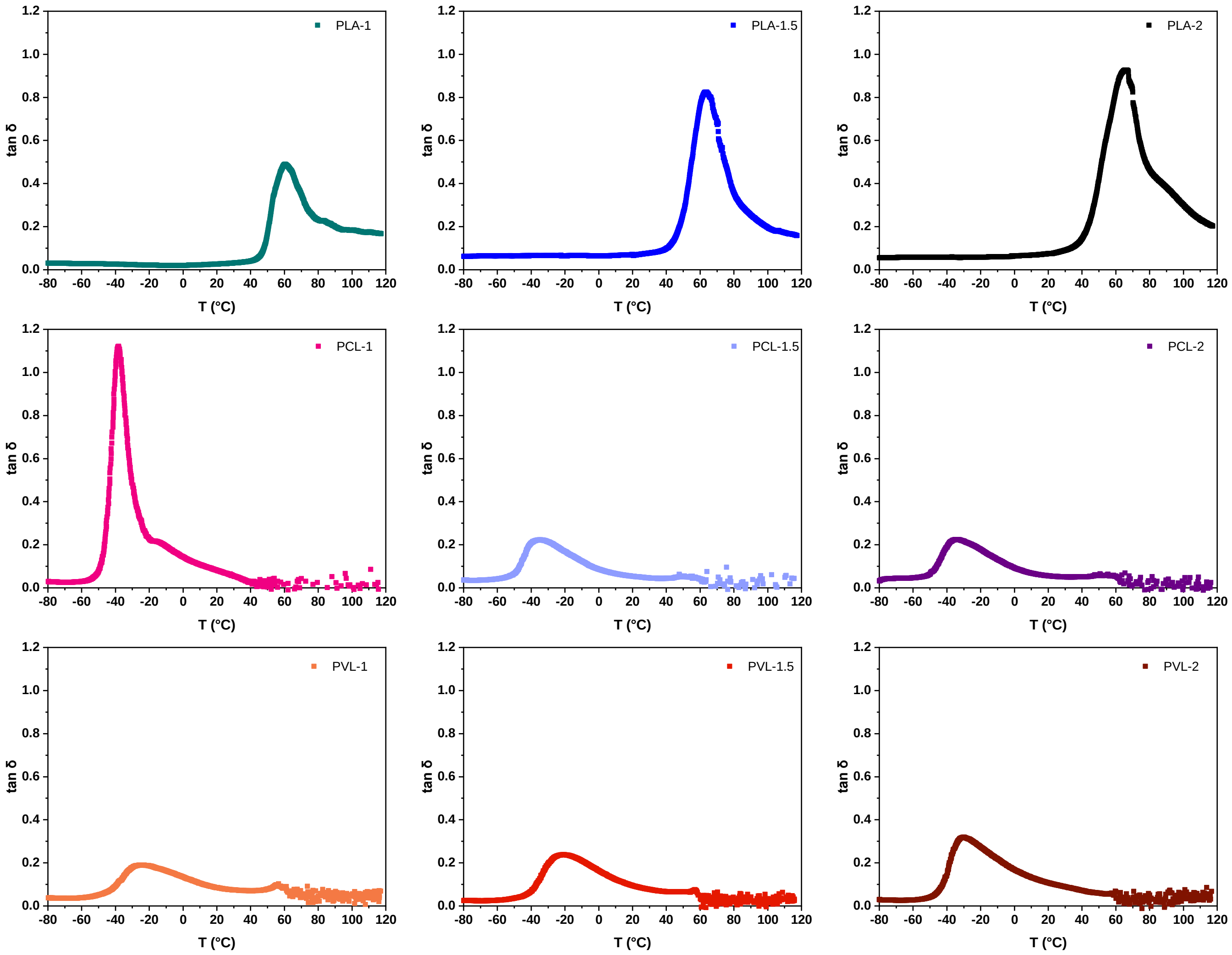

Figure S17. Tan δ curves plotted for all cross-linked polyesters synthesised.

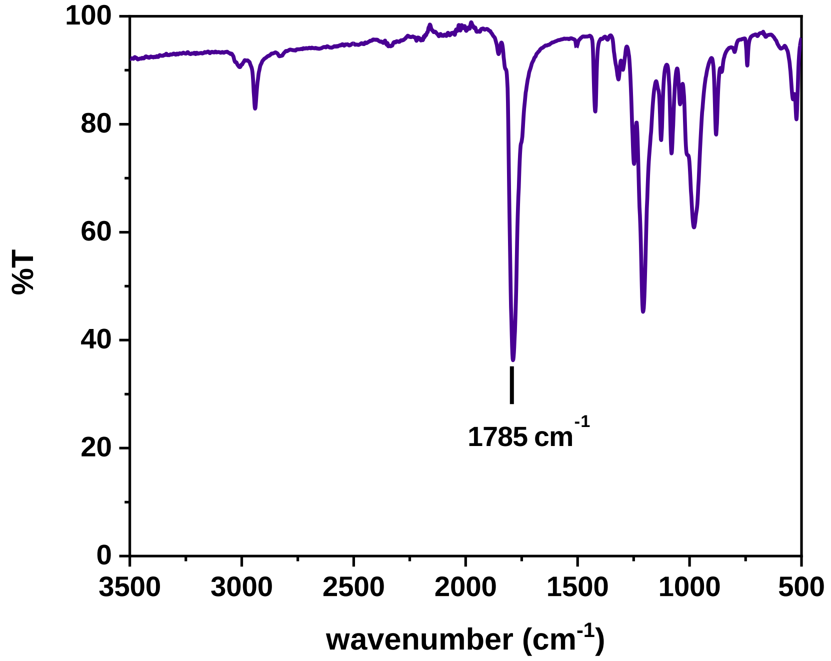

Figure S18. FTIR spectrum (neat) of **bisDOX**, with its characteristic carbonyl stretching frequency labelled at 1785 cm^{-1}.

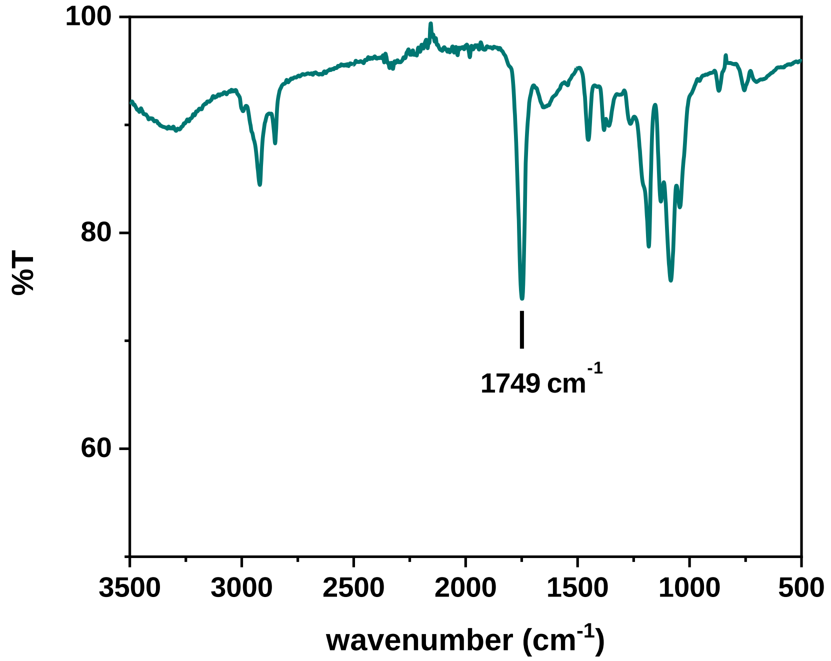

Figure S19. FTIR spectrum (neat) of **PLA-1**, with its characteristic carbonyl stretching frequency labelled at 1749 cm^{-1}.

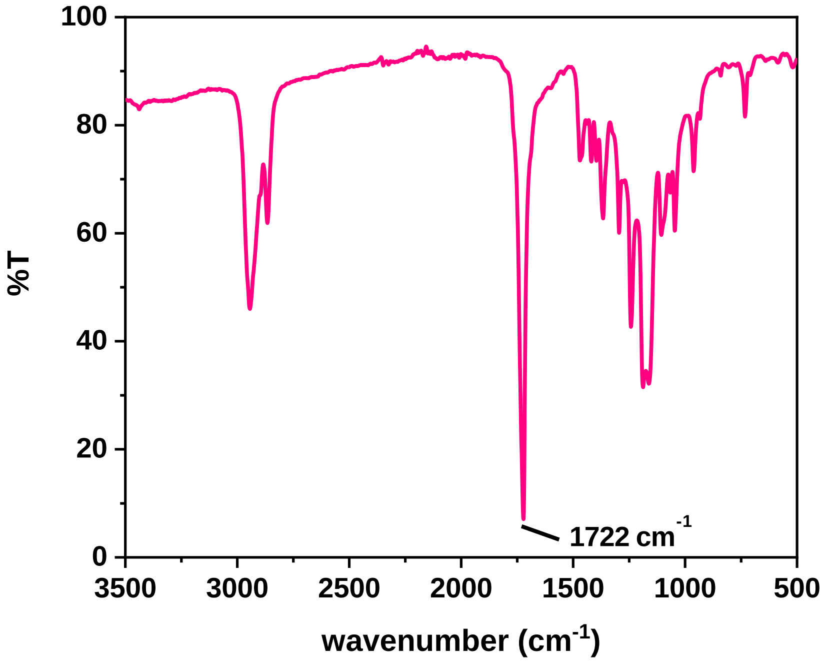

Figure S20. FTIR spectrum (neat) of **PCL-1**, with its characteristic carbonyl stretching frequency labelled at 1722 cm^{-1}.

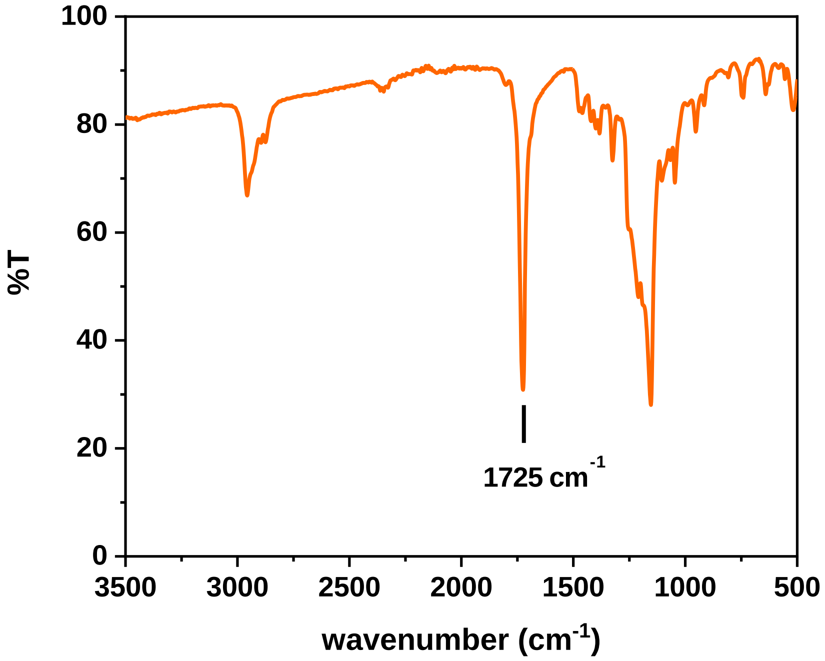

Figure S21. FTIR spectrum (neat) of **PVL-1**, with its characteristic carbonyl stretching frequency labelled at 1725 cm^{-1}.

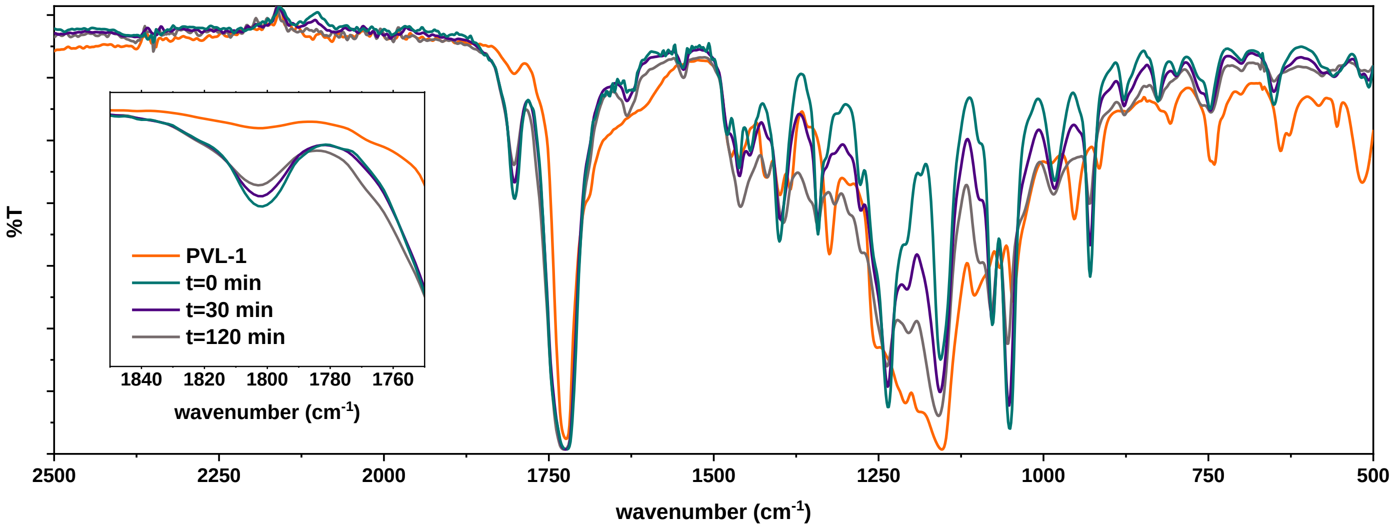

Figure S22. FTIR spectra (neat) of **PVL-1** reaction mixture before cross-linking (t = 0 min), during cross-linking (t = 30 min, t = 120 min) and after cross-linking (**PVL-1**), with an inset showing the carbonyl stretching frequency of **bisDOX** disappearing as the reaction progresses.

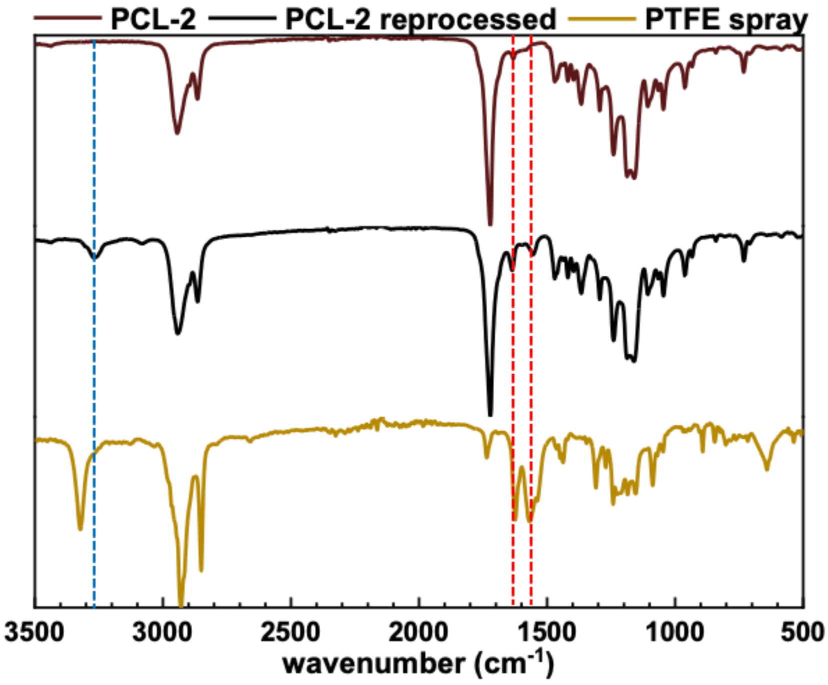

Figure S23. FTIR spectra (neat) of **PCL-2** before and after reprocessing. A third spectrum, that of the PTFE mould release spray, is also included. There are three obvious peaks appearing post-reprocessing, indicated by the dotted lines. There is a possibility the two peaks labelled by the red dotted line can be ascribed to the release spray that aids seamless removal of the resins from the moulds. The third peak, indicated by the blue line could correspond to a N–H stretching vibration. While a similar peak is present in the mould release spray spectra, the mismatch suggests the peak could arise from decomposition of the mould release spray formulation or from the hydrolytic degradation of the salen catalyst liberating an amine.

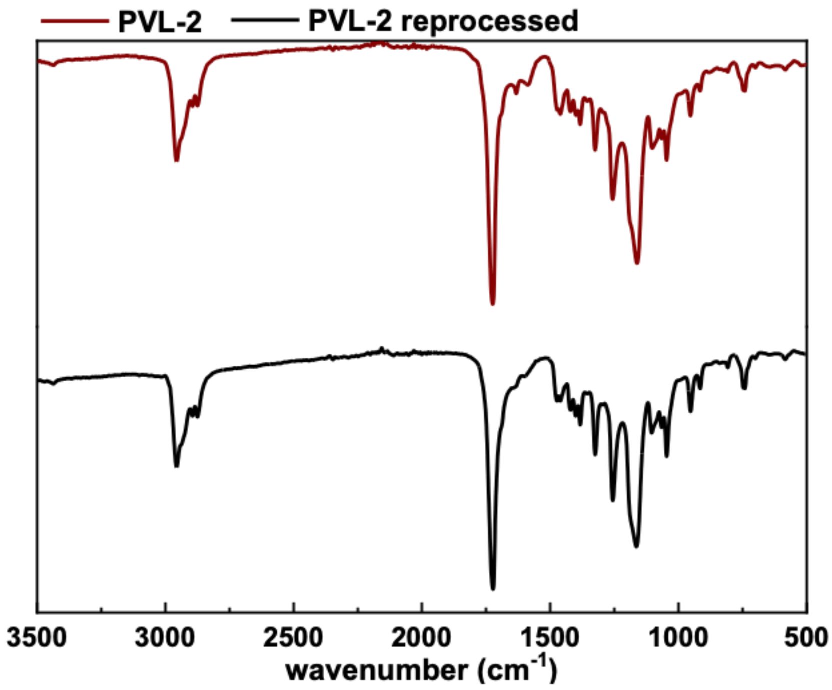

Figure S24. FTIR spectra (neat) of **PVL-2** before and after reprocessing.

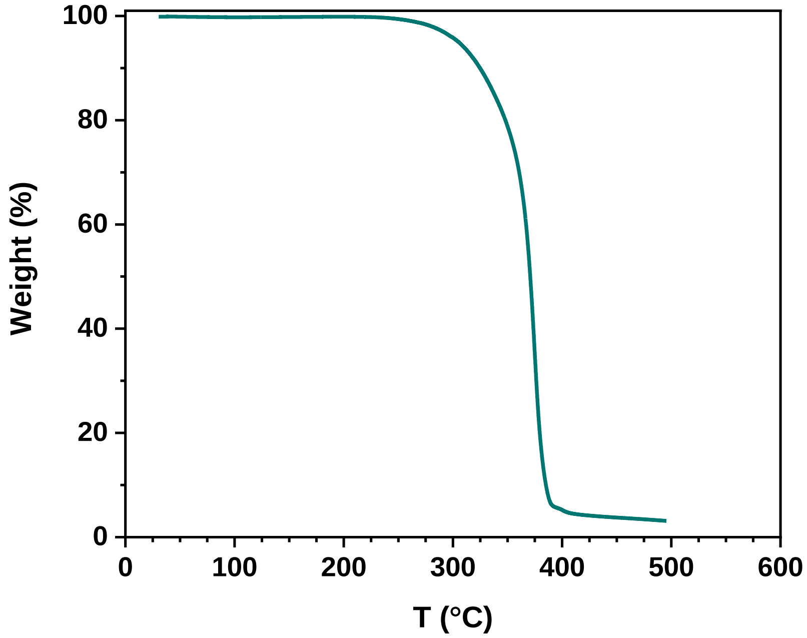

Figure S25. Thermogravimetric analysis (TGA) of **PLA-1**.

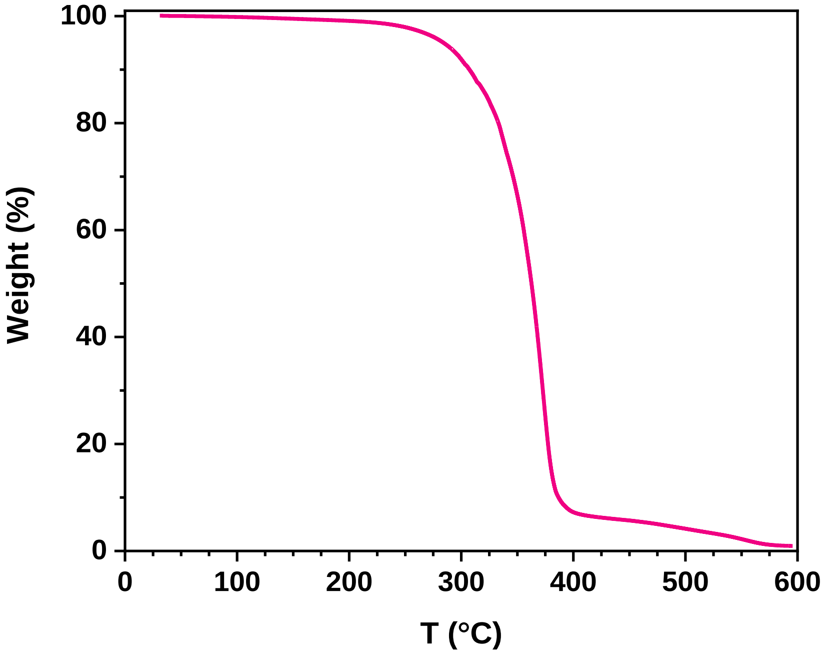

Figure S26. Thermogravimetric analysis (TGA) of **PCL-1**.

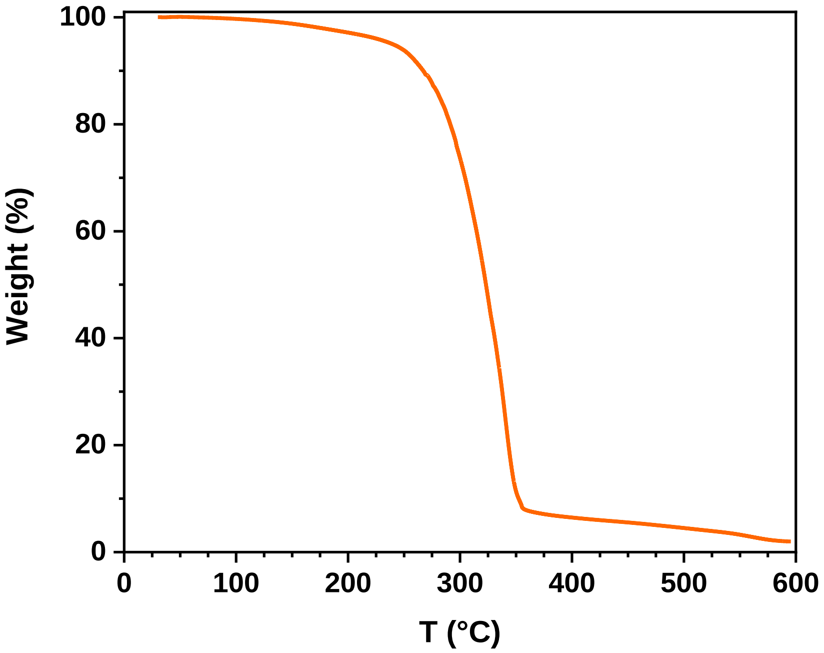

Figure S27. Thermogravimetric analysis (TGA) of **PVL-1.**

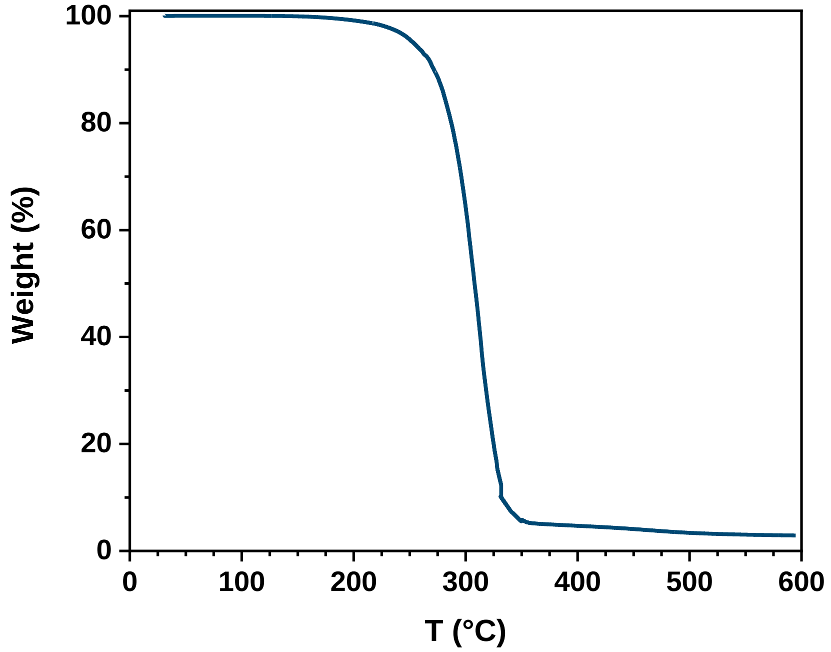

Figure S28. Thermogravimetric analysis (TGA) of **PLA-1.5**.

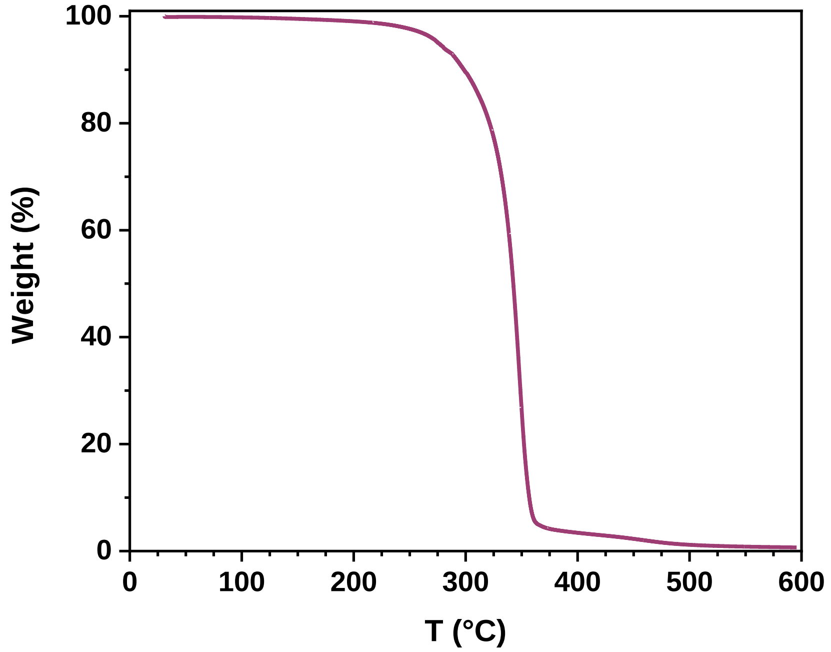

Figure S29. Thermogravimetric analysis (TGA) of **PCL-1.5**.

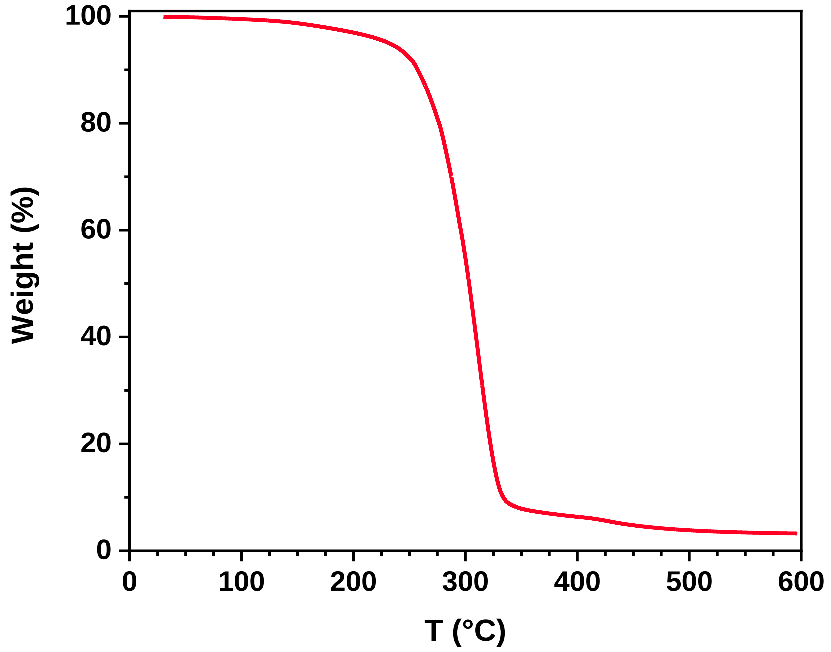

Figure S30. Thermogravimetric analysis (TGA) of **PVL-1.5**.

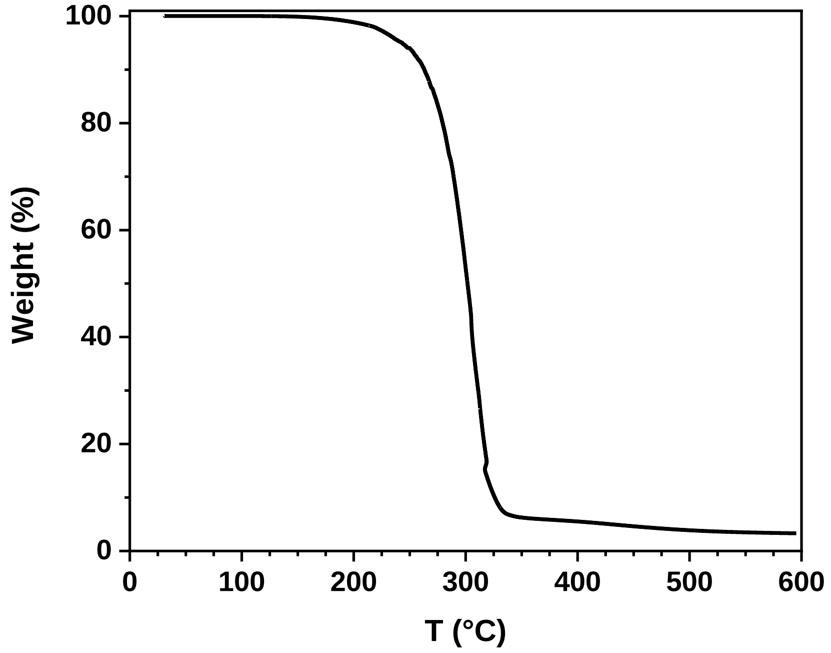

Figure S31. Thermogravimetric analysis (TGA) of **PLA-2**.

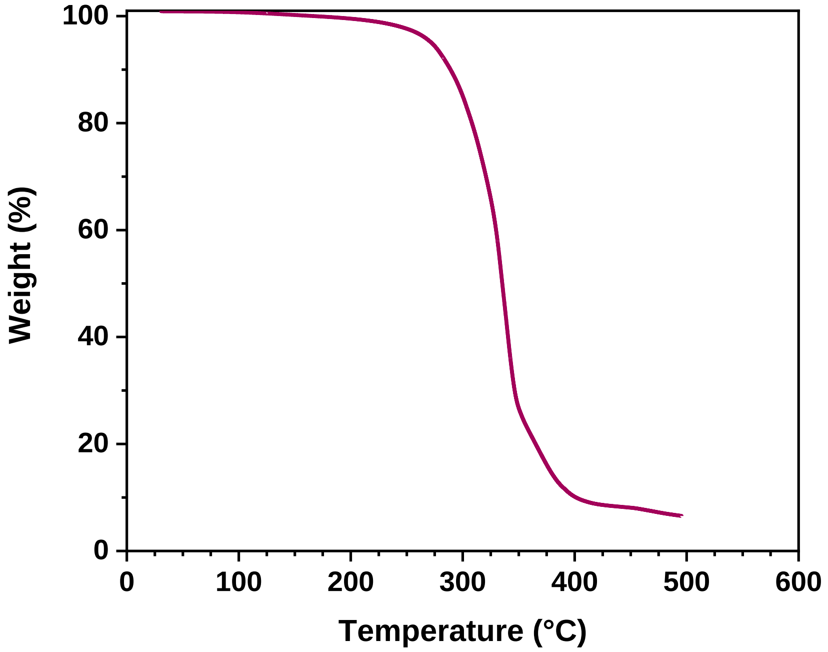

Figure S32. Thermogravimetric analysis (TGA) of **PCL-2.**

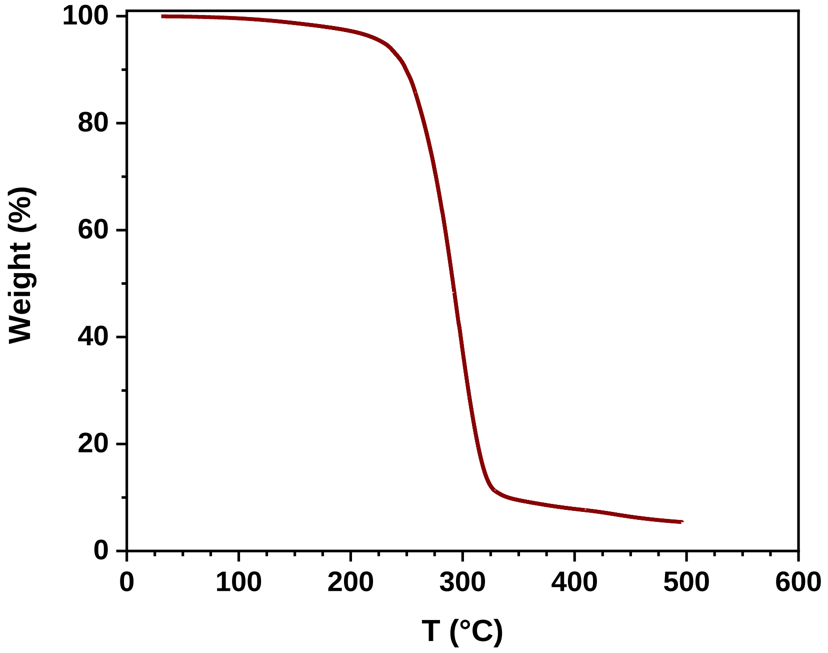

Figure S33. Thermogravimetric analysis (TGA) for **PVL-2**.

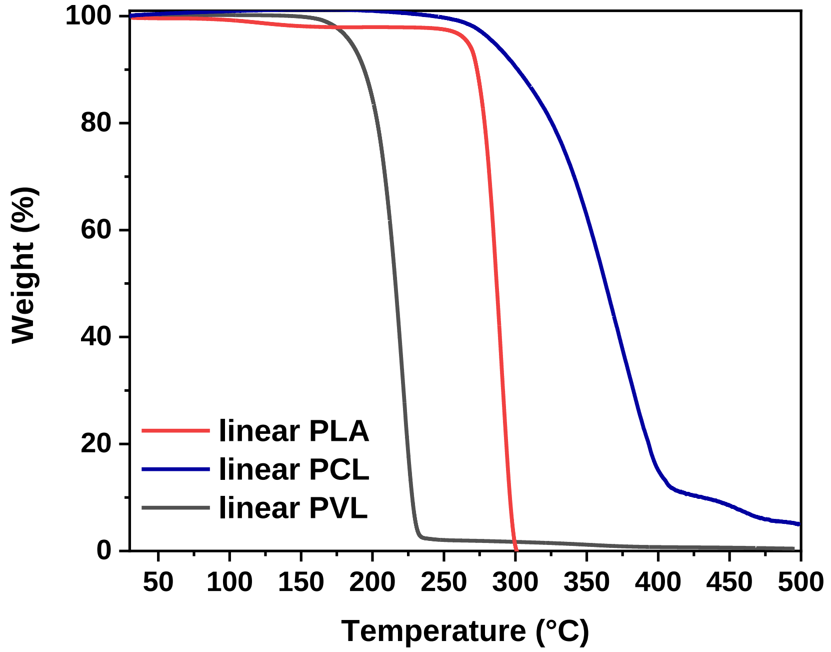

Figure S34. Thermogravimetric analysis (TGA) for PLA, PCL and PVL homopolymers.

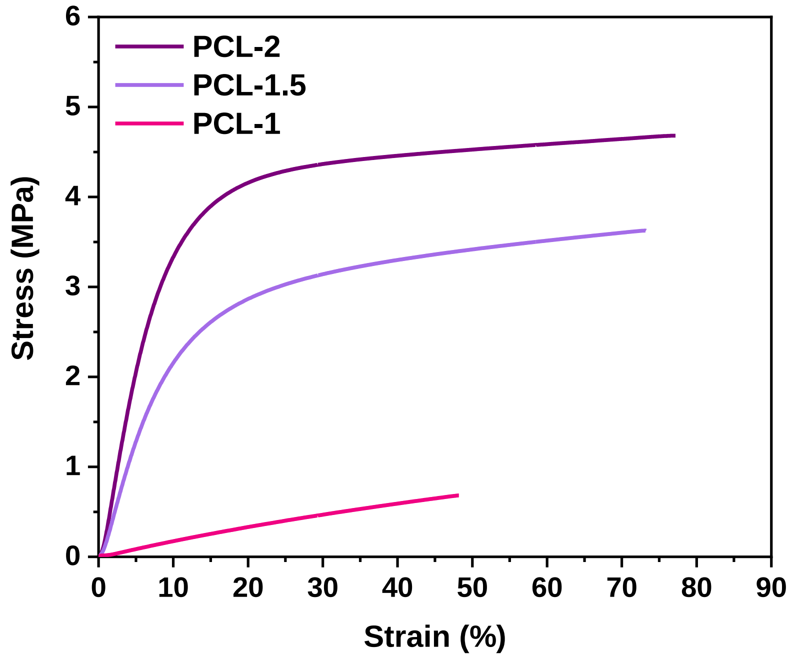

Figure S35. Stress-strain curves for **PCL-1**, **PCL-1.5** and **PCL-2**.

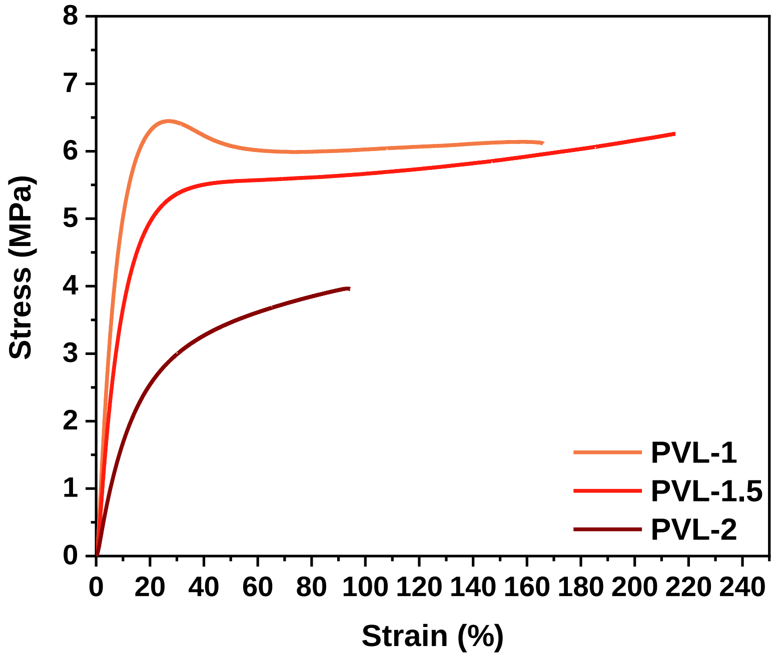

Figure S36. Stress-strain curves for **PVL-1**, **PVL-1.5** and **PVL-2**.

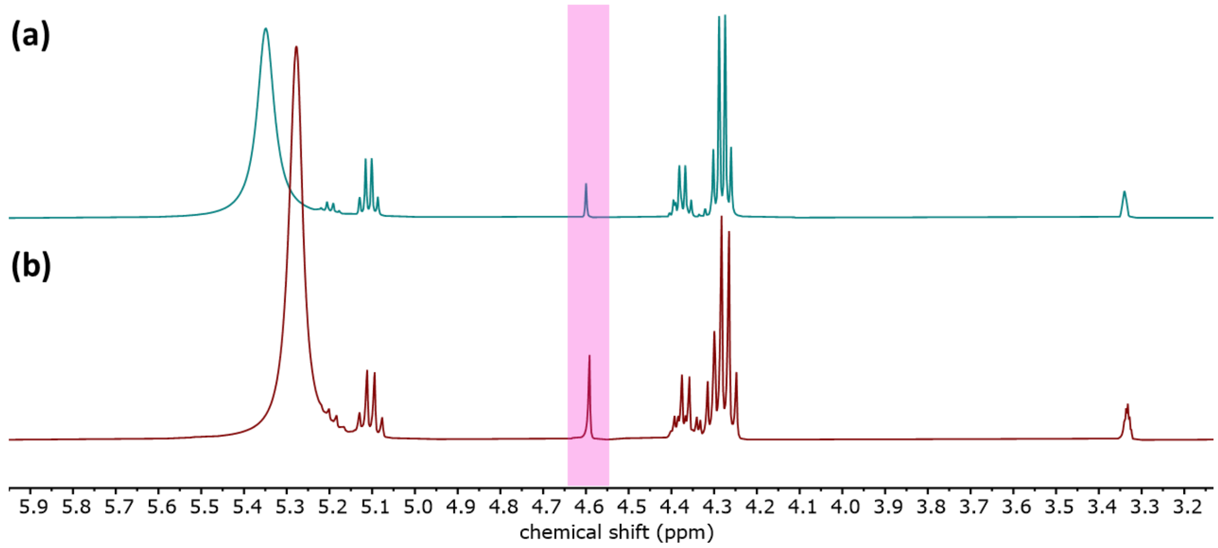

Figure S37. ^{1}H NMR spectra (CD_3OD, 500 MHz) (a) **PLA-1** degradation products (b) **PLA-1** degradation products spiked with L-(+)-tartaric acid. The L-(+)-tartaric acid resonance is highlighted in magenta.

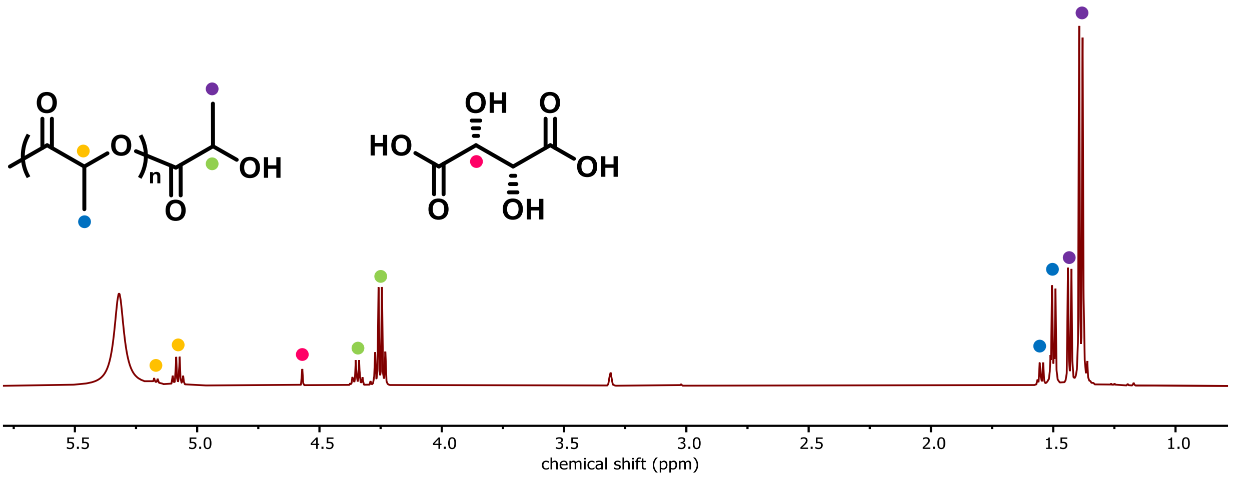

Figure S38. ^{1}H NMR spectra (CD_3OD, 500 MHz) **PLA-1** degradation products.

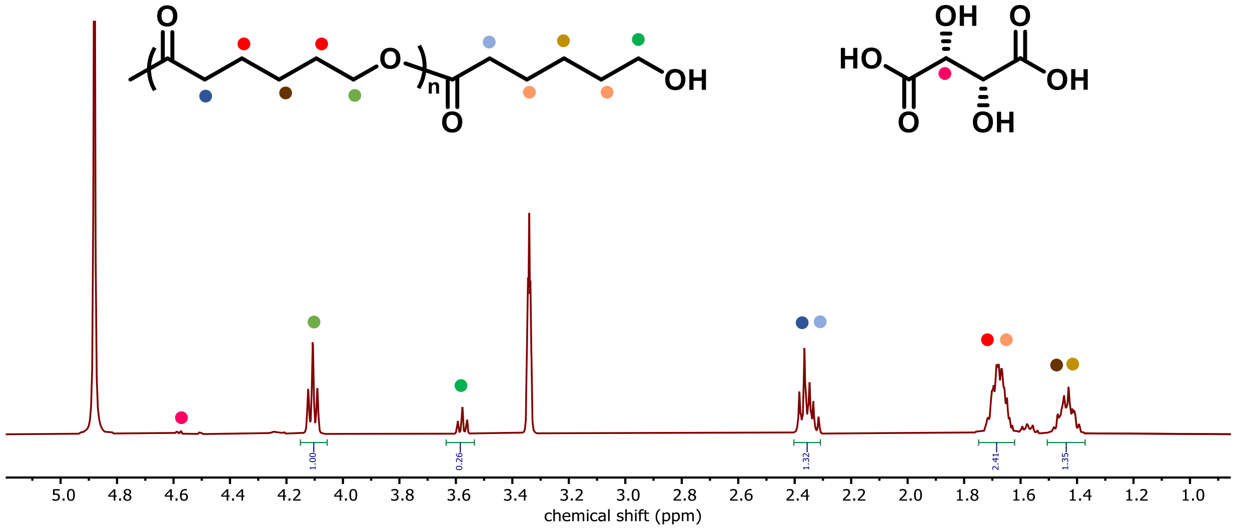

Figure S39. ^{1}H NMR spectra (CD_3OD, 500 MHz) **PCL-1** degradation products.

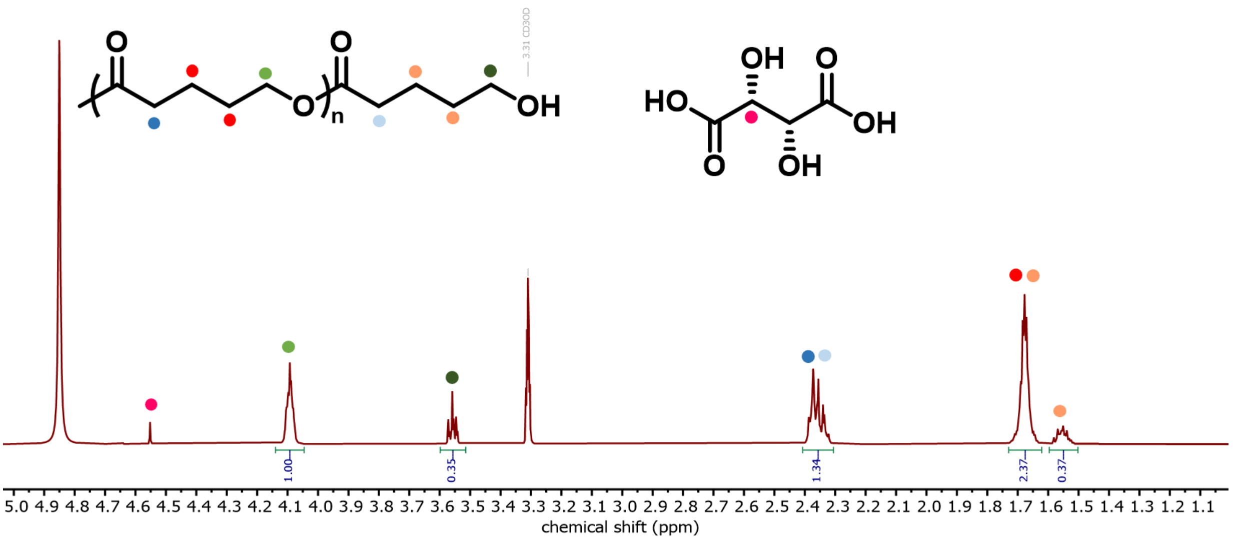

Figure S40. ^{1}H NMR spectra (CD_3OD, 500 MHz) **PVL-1** degradation products.

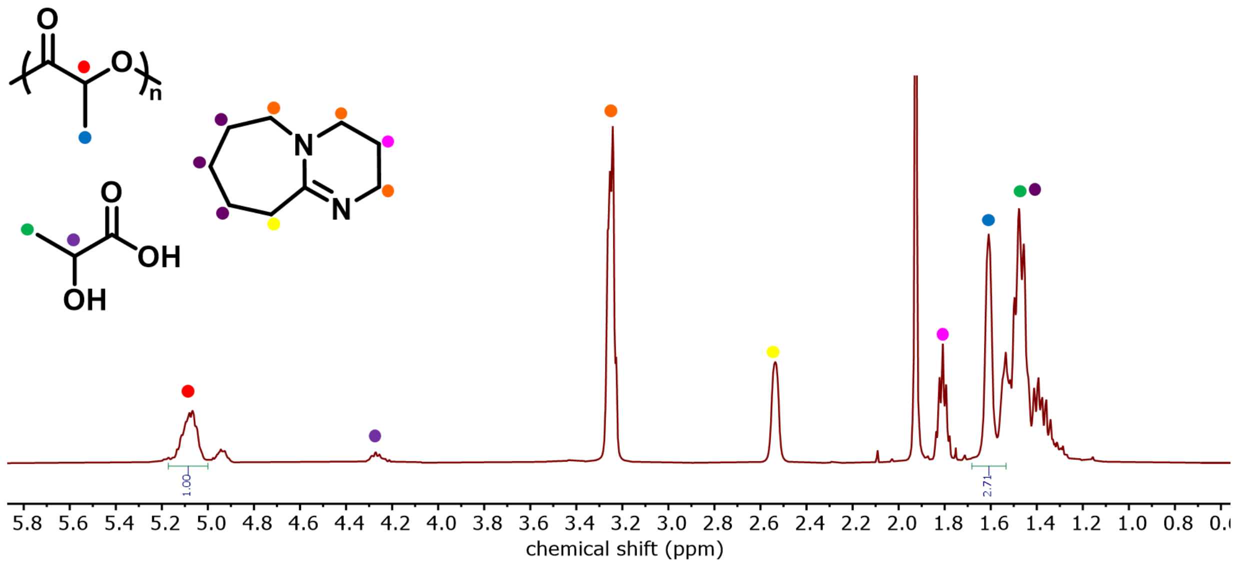

Figure S41. ^{1}H NMR spectrum ($CDCl_3$, 500 MHz) of **PLA-1** degraded in DBU/acetonitrile, along with ^{1}H assignments of reaction mixture components.

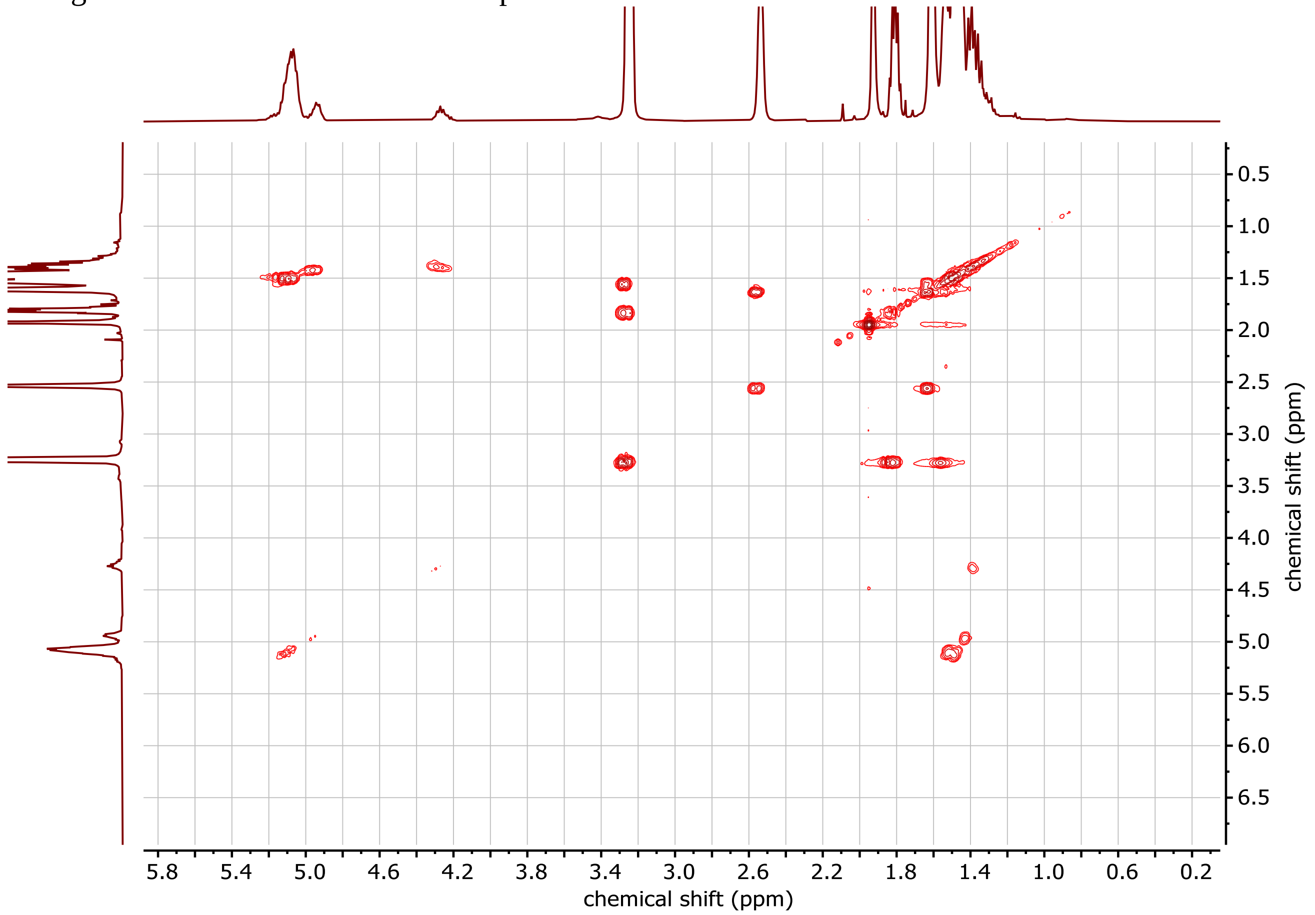

Figure S42. ^{1}H-^{1}H COSY NMR spectrum ($CDCl_3$, 500 MHz) of **PLA-1** degraded in DBU/acetonitrile.

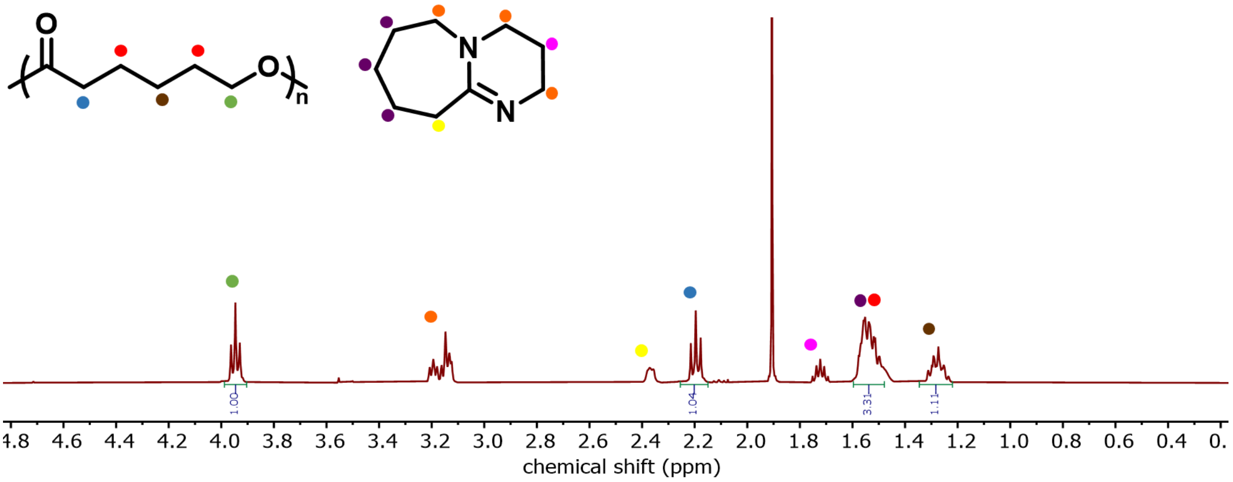

Figure S43. ^1H NMR spectrum ($CDCl_3$, 500 MHz) of **PCL-1** degraded in DBU/acetonitrile, along with ^1H assignments of reaction mixture components.

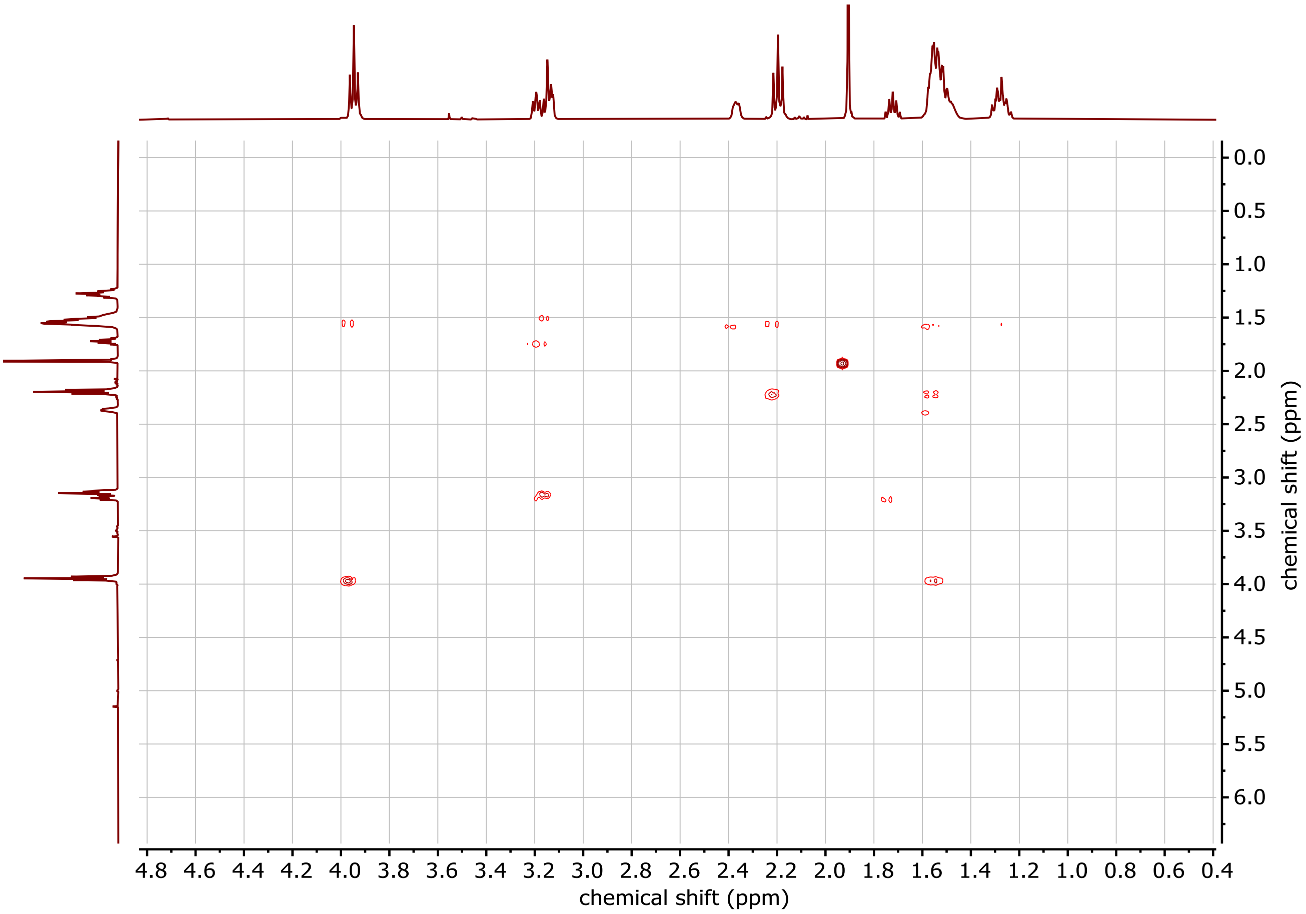

Figure S44. ^1H-^1H COSY NMR spectrum ($CDCl_3$, 500 MHz) of **PCL-1** degraded in DBU/acetonitrile.

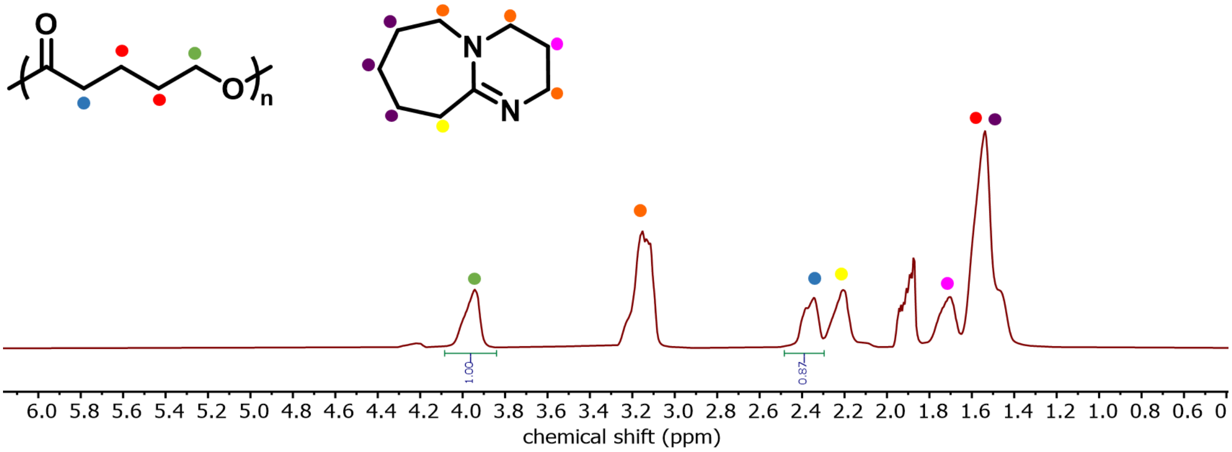

Figure S45. ^1H NMR spectrum ($CDCl_3$, 500 MHz) of **PVL-1** degraded in DBU/acetonitrile, along with ^1H assignments of reaction mixture components.

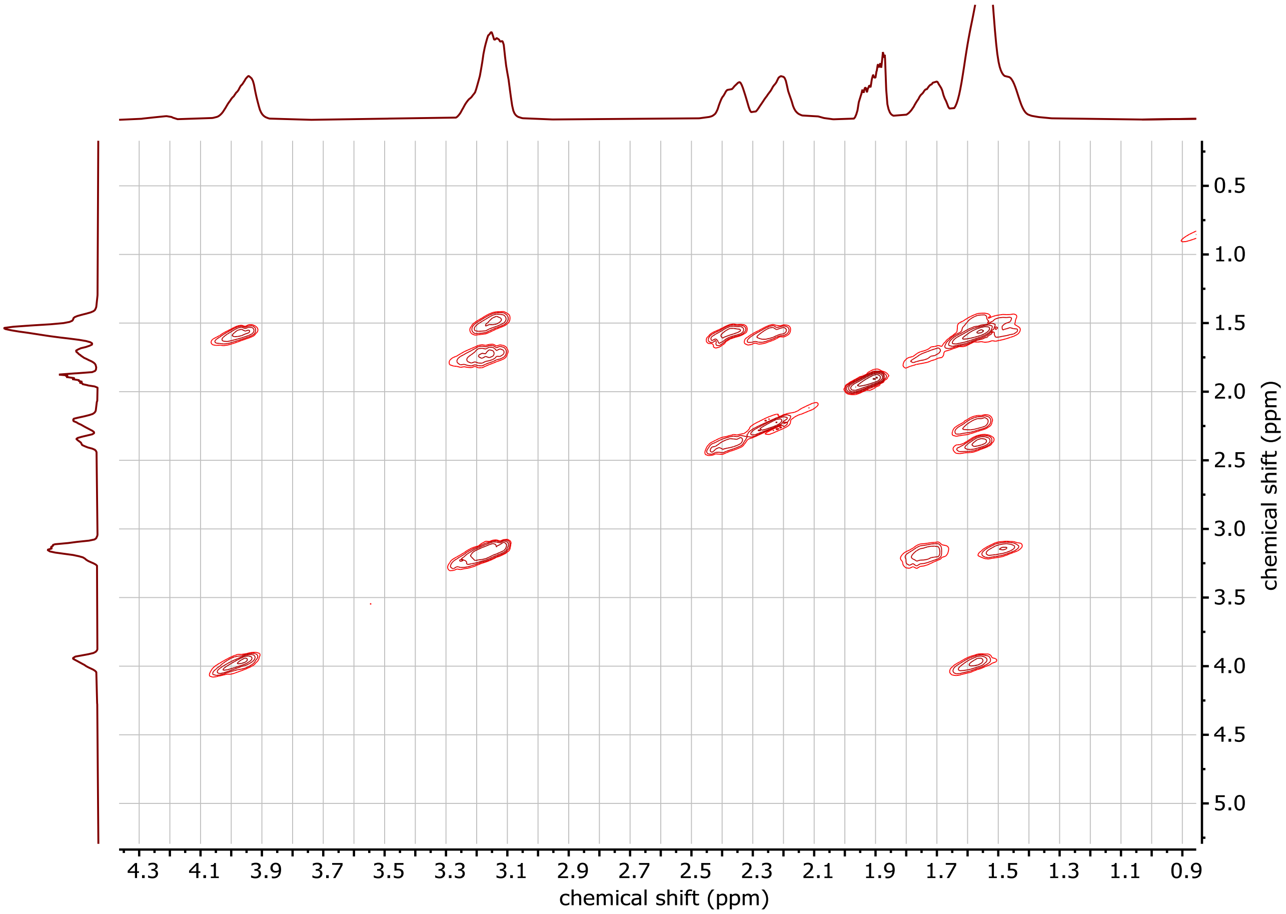

Figure S46. ^1H-^1H COSY NMR spectrum ($CDCl_3$, 500 MHz) of **PVL-1** degraded in DBU/acetonitrile.

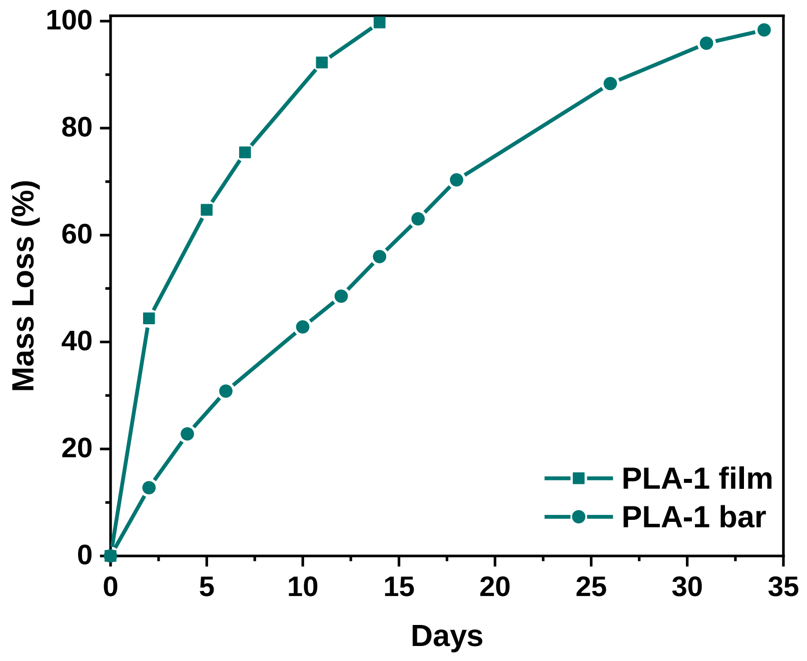

Figure S47. Degradation profiles for **PLA-1-film** (40 × 4 × 1 mm) and **PLA-1-bar** (60×12×3 mm) in aqueous 1 M NaOH solution.

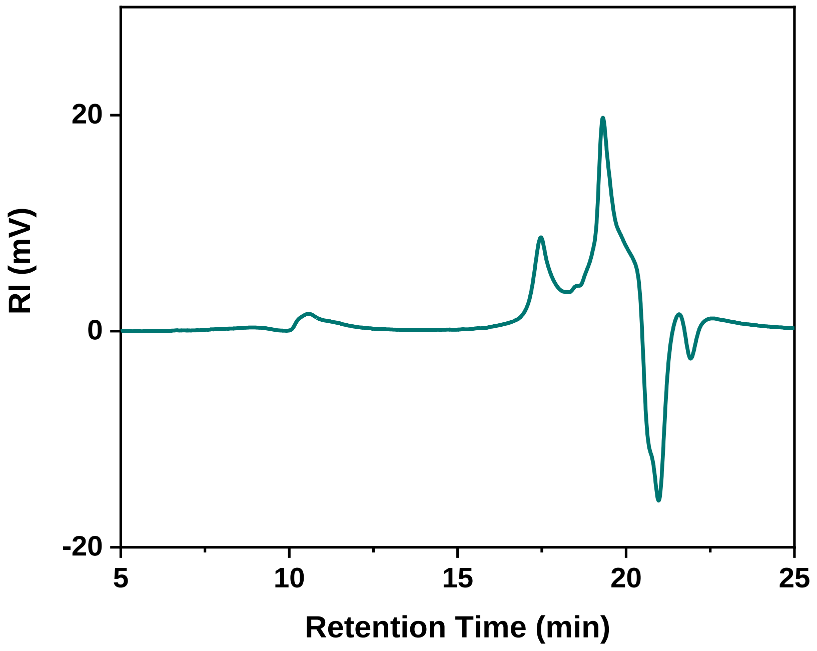

Figure S48. GPC trace of **PLA-1** degradation products (NaOH).

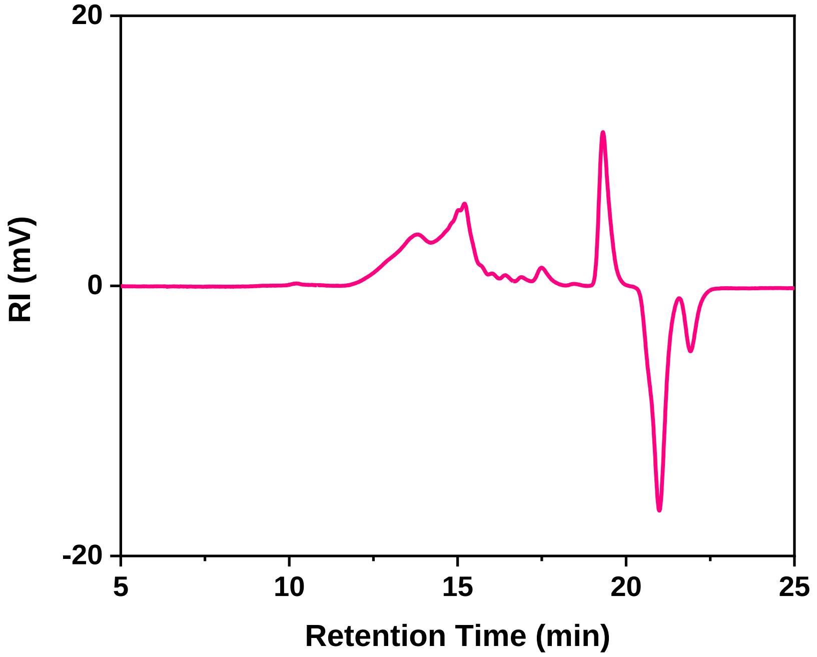

Figure S49. GPC trace of **PCL-1** degradation products (NaOH).

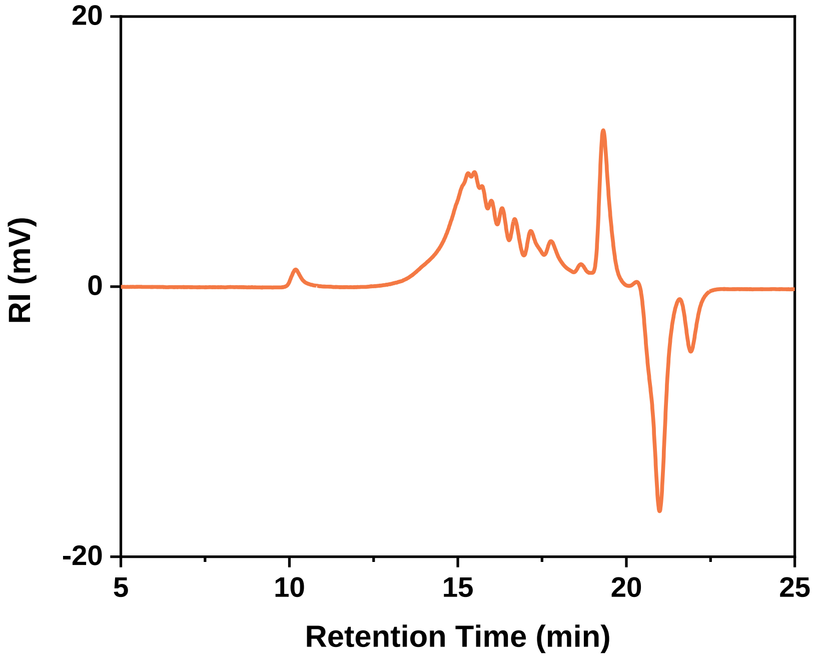

Figure S50. GPC trace of **PVL-1** degradation products (NaOH).

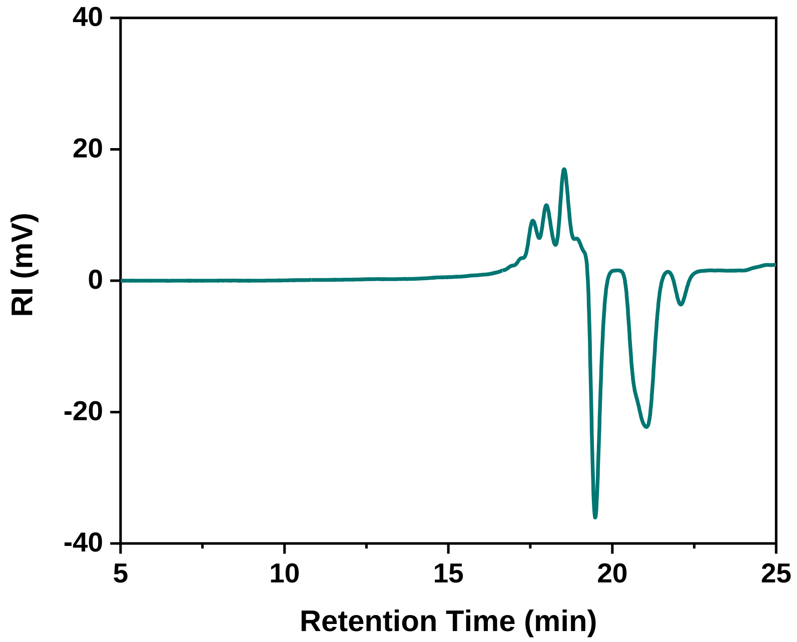

Figure S51. GPC trace of **PLA-1** degradation products (DBU/acetonitrile).

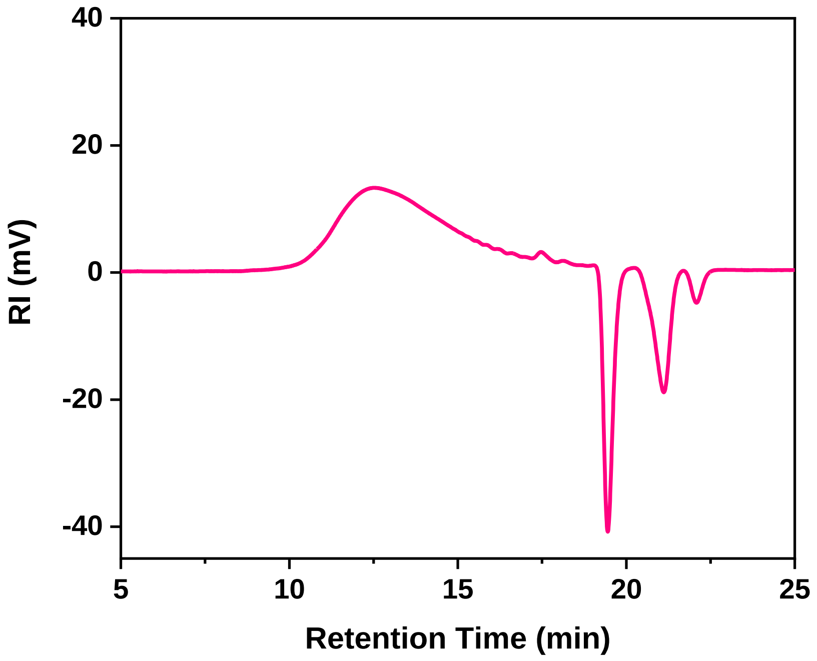

Figure S52. GPC trace of **PCL-1** degradation products (DBU/acetonitrile).

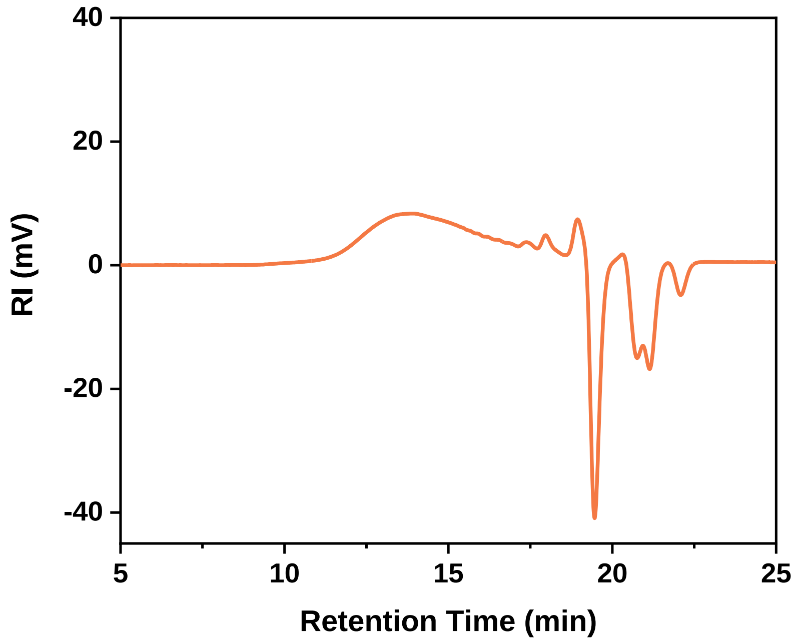

Figure S53. GPC trace of **PVL-1** degradation products (DBU/acetonitrile).

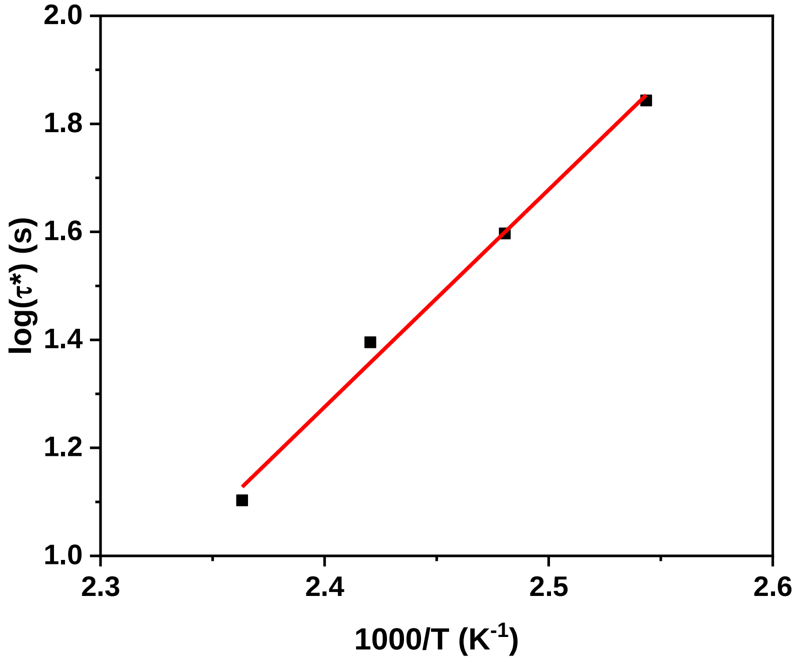

Figure S54. Arrhenius plot relating the characteristic relaxation time (τ*) to inverse temperature for **PVL-2** along with a fitted line with R^2=0.99273.

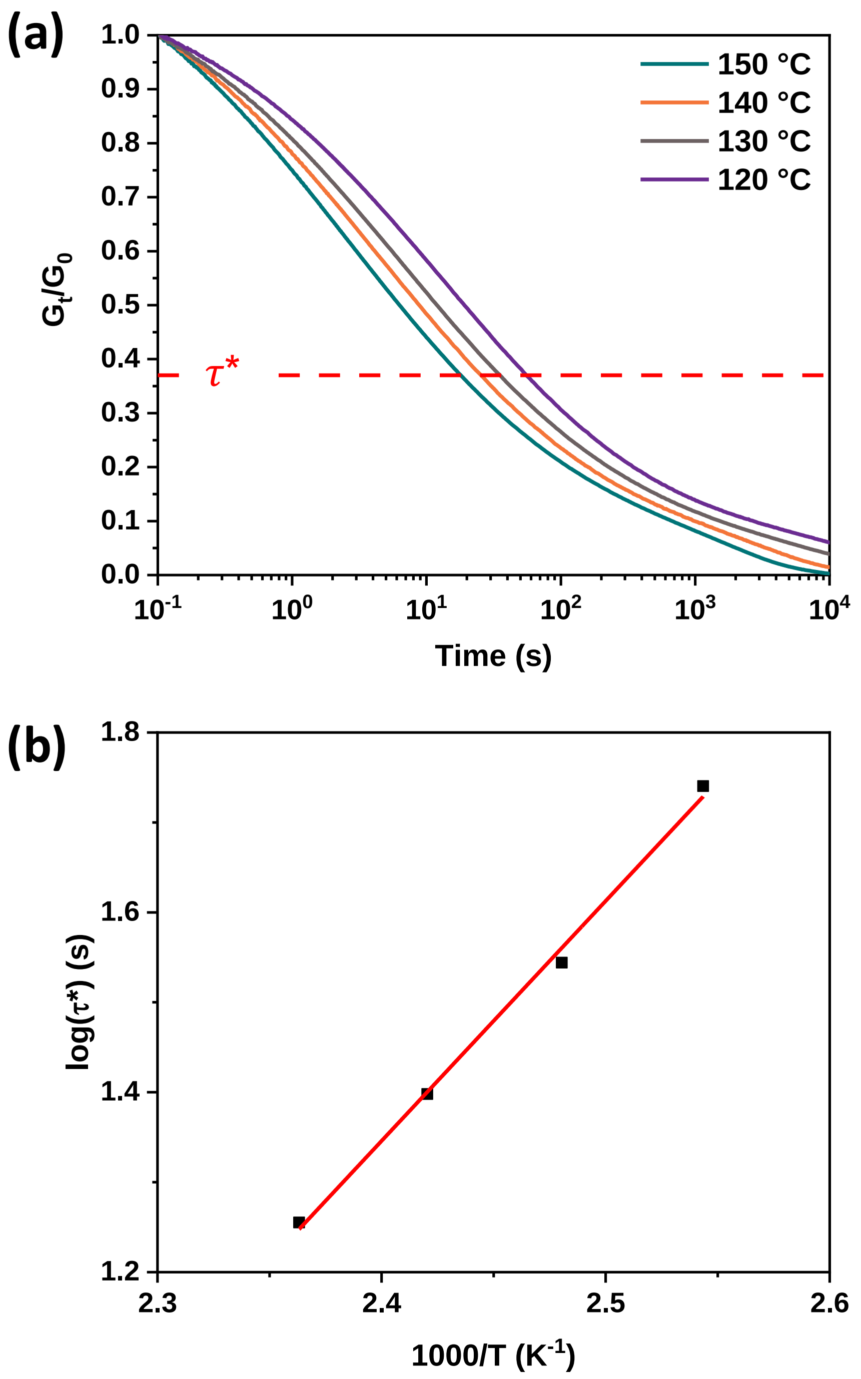

Figure S55. (a) Stress relaxation curves of polymer **PVL-1.5**. (b) Arrhenius plot relating the characteristic relaxation time (τ*) to inverse temperature for **PVL-1.5** along with a fitted line with R^2=0.99639.

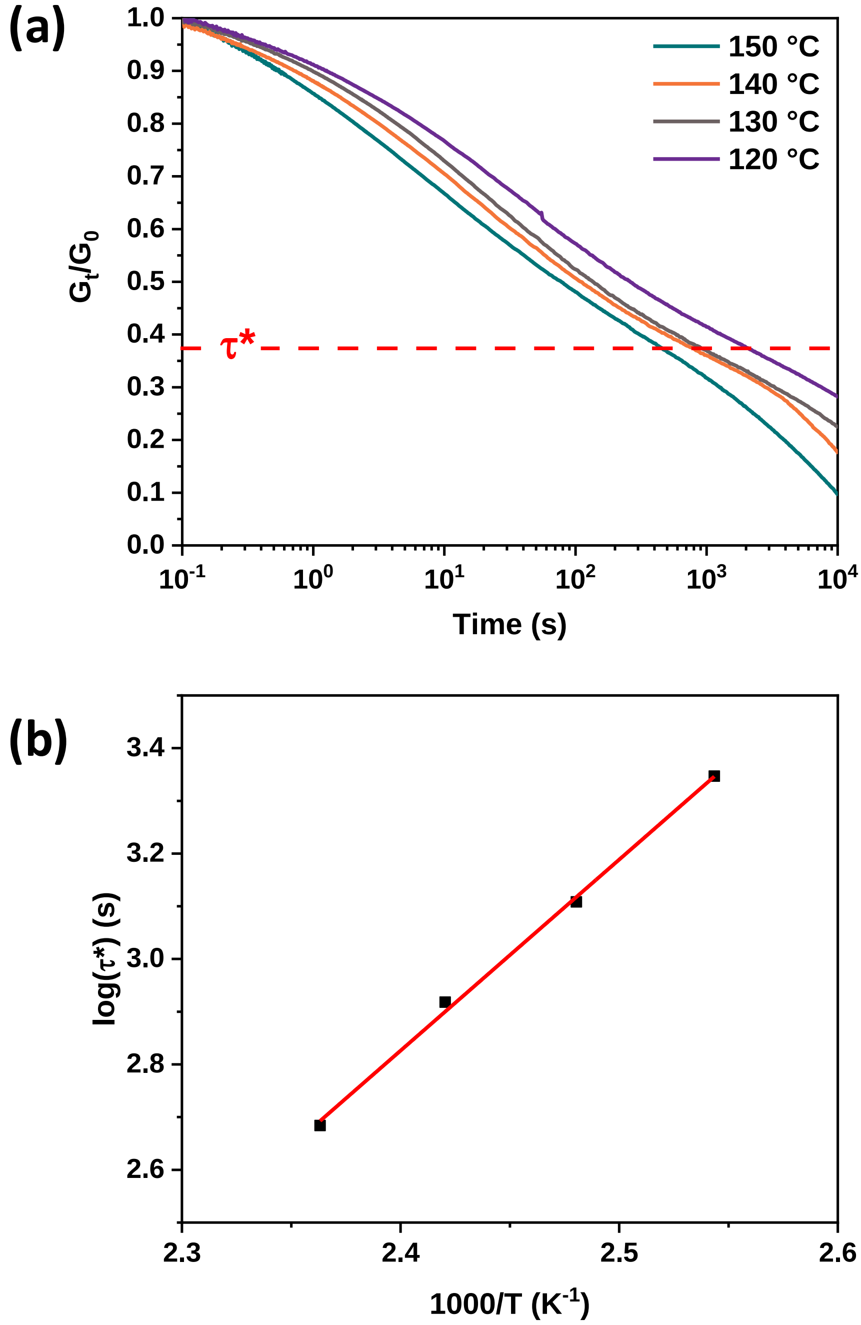

Figure S56. (a) Stress relaxation curves of polymer **PCL-1.5**. (b) Arrhenius plot relating the characteristic relaxation time (τ*) to inverse temperature for **PCL-1.5** along with a fitted line with R^2=0.99897.

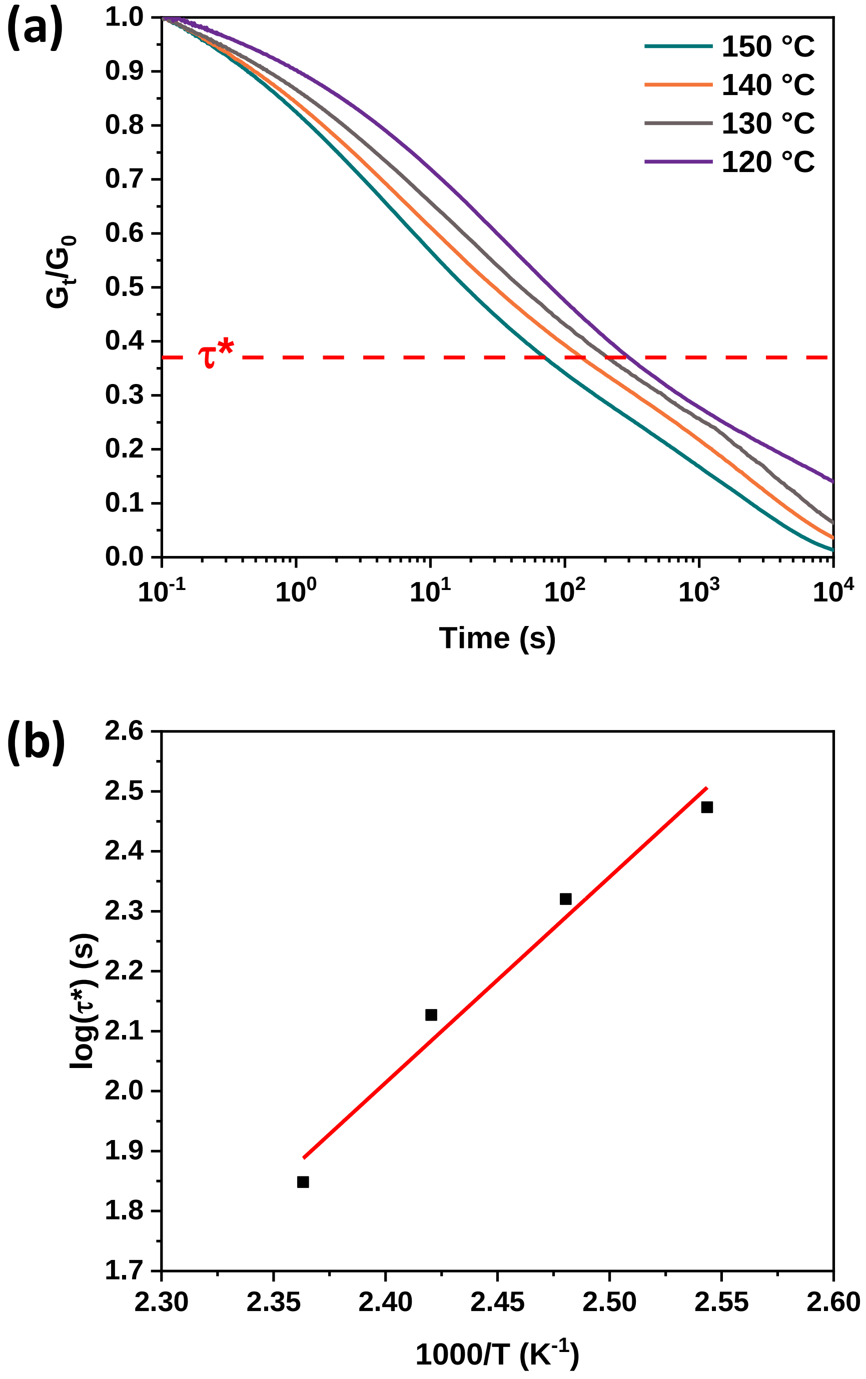

Figure S57. (a) Stress relaxation curves of polymer **PCL-2**. (b) Arrhenius plot relating the characteristic relaxation time (τ*) to inverse temperature for **PCL-2** along with a fitted line with R^2=0.99095.

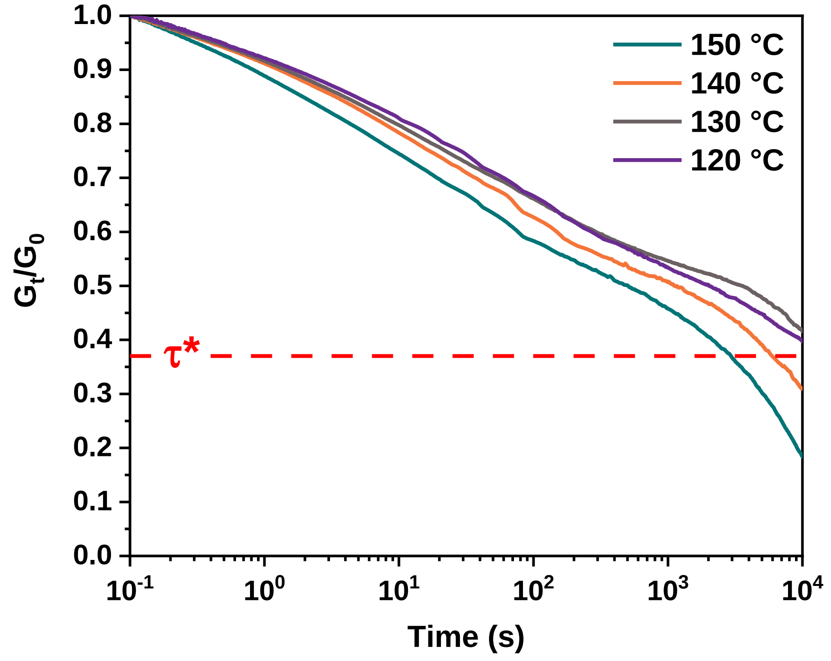

Figure S58. Stress relaxation curves of polymer **PCL-1**.

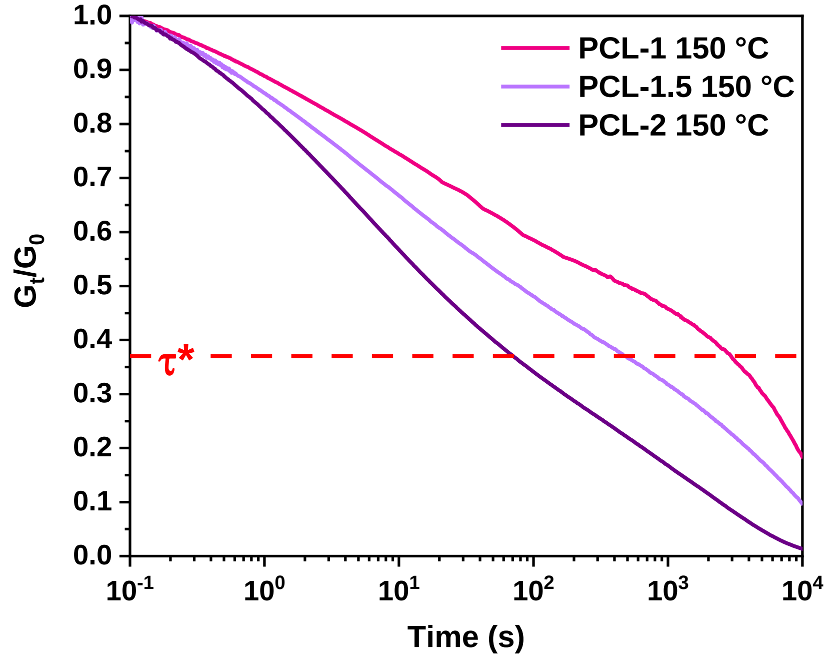

Figure S59. Stress relaxation curves of polymers **PCL-1**, **PCL-1.5**, and **PCL-2** at 150 °C.

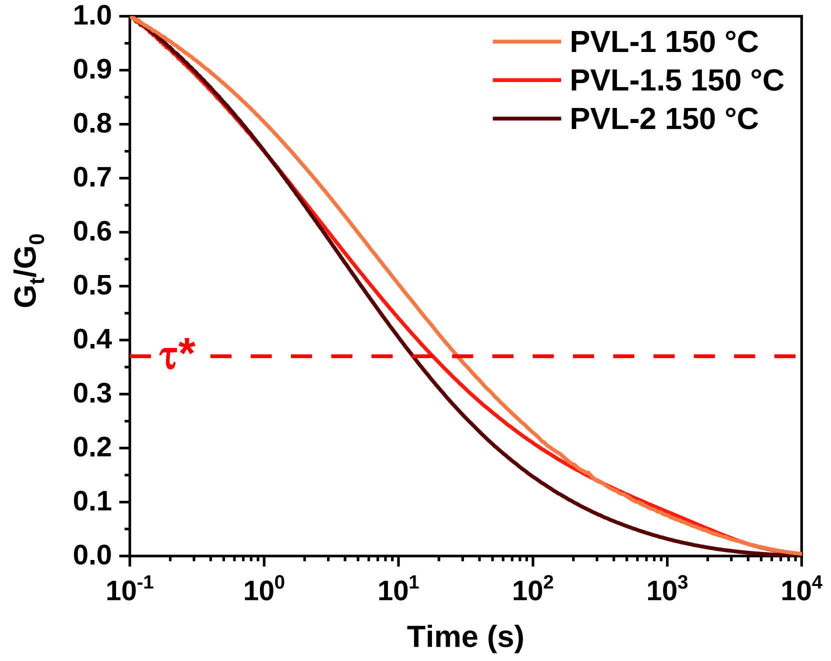

Figure S60. Stress relaxation curves of polymers **PVL-1**, **PVL-1.5**, and **PVL-2** at 150 °C.

Table S1. Reaction optimization performed in toluene (5 M monomer concentration) for copolymerizations between bisDOX and L-lactide or ε-caprolactone.

bisDOX mol %	Observation
0.63	did not set, viscous liquid
1	did not set, viscous liquid
1.25	did not set, viscous liquid
2	very viscous liquid
2.5	very viscous liquid
3	gel, high soluble fractions, homopolymer identified in ^{1}H NMR spectrum of extracts
4	gel, high soluble fractions, homopolymer identified in ^{1}H NMR spectrum of extracts
5	gel, minimum amount of unreacted monomers compared to other formulations
7.5	gel, low yielding insoluble material, unreacted bisDOX and cyclic ester recovered and identified following swelling in $CDCl_3$ and subsequent NMR spectroscopic analysis
10	gel, along with low yielding insoluble material, unreacted bisDOX and cyclic ester recovered and identified following swelling in $CDCl_3$ and subsequent NMR spectroscopic analysis
50	viscous liquid, low yielding insoluble material, unreacted bisDOX and cyclic ester recovered and identified following swelling in $CDCl_3$ and subsequent NMR spectroscopic analysis

Table S2. Thermal and mechanical properties of our synthesized thermosets with varying catalyst loadings.

Sample	Gel %	T_g (°C)[a]	T_m (°C)[a]	ΔH_m (J/g)[a]	T_c (°C)[b]	ΔH_c (J/g)[b]	T_{cc} (°C)[a]	ΔH_{cc} (J/g)[a]	$T_{d,5\%}$ (°C)[c]	σ_b (MPa)[d]	ε_b (%)[d]	E (MPa)[d]
PLA-1	95	62.7	-	-	-	-	-	-	305	46.7±3.4	4.5±0.4	1268±43
PLA-1.5	95	34.3	140.5	11.1	-	-	-	-	254	n/a	n/a	n/a
PLA-2	90	32.5	139.2	10.6	-	-	-	-	243	n/a	n/a	n/a
PCL-1	92	−57.0	24.0	23.8	−30.1	2.0	−18.0	22.3	270	1.1±0.1	83±11	2.2±0.4
PCL-1.5	89	−54.9	33.3	36.6	−9.0	29.4	−22.4	3.7	276	3.7±0.2	71±12	30.5±2.9
PCL-2	85	−52.7	34.8	38.7	−7.4	34.1	−23.2	1.0	272	5.0±0.1	74±8	51.9±3.3
PVL-1	79	−51.3	36.4	40.0	−12.8	29.7	−21.1	1.8	260	5.9±0.3	147±82	67.3±2.6
PVL-1.5	79	−56.6	34.4	35.3	−18.8	16.3	−21.1	11.8	232	5.9±0.4	205±37	47.2±7.3
PVL-2	88	−56.4	31.2	29.4	−23.6	4.6	−15.8	23.1	230	3.8±0.2	94±11	19.0±3.0

[a] Data obtained from the second heating ramp of a DSC experiment; [b] Data obtained from the first cooling ramp of a DSC experiment; [c] Data obtained from TGA experiments; [d] Data obtained from tensile testing measurements of at least five replicates.

Table S3. Thermomechanical characterization of the cross-linked resins.

Sample	E' at 100 °C (MPa)	ν_e (10^{-4} mol cm^{-3})[a,b]	M_x (kg mol^{-1})[a,b]
PLA-1	49.2	52.9	0.24
PLA-1.5	33.5	36	0.35
PLA-2	8.5	9.1	1.4
PCL-1	3.80	0.41	9.39
PCL-1.5	3.00	0.32	11.83
PCL-2	2.52	0.27	14.10
PVL-1	0.92	0.10	38.69
PVL-1.5	1.08	0.12	32.73
PVL-2	1.23	0.13	28.74

[a] Data obtained using Equation (1) at 100 ºC; [b] Data obtained based on a density ρ=1.25 g cm^{-1} for PLA,[2] ρ=1.145 g cm^{-3} for PCL,[3] and ρ=1.14 g cm^{-3} for PVL.[4]

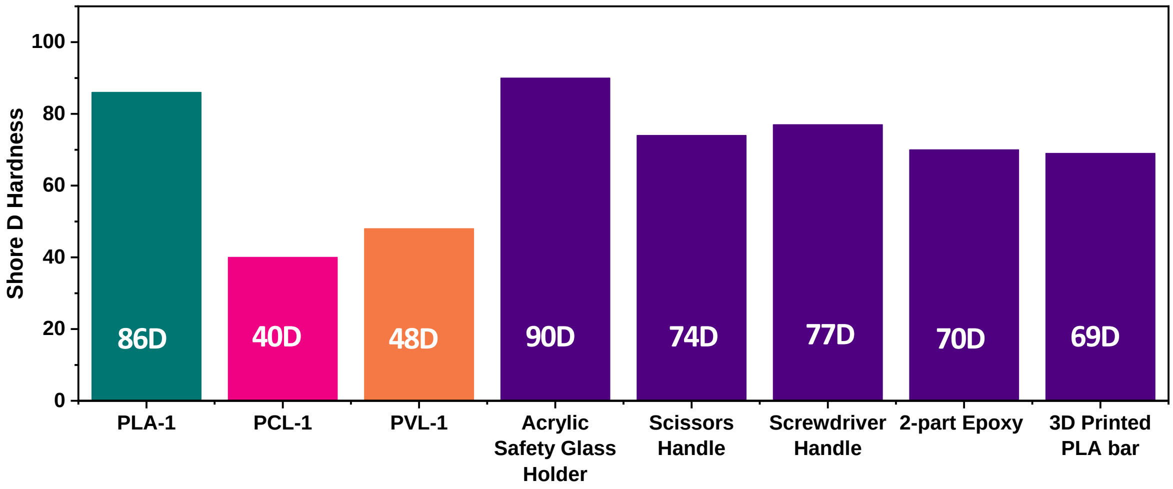

Figure S61. Shore D hardness values for a variety of objects tested.

Table S4. Degradation products investigated by GPC analysis.

**Sample (Degradation Medium)**	M_n **(g mol**$^{-1}$**)**	***Đ***	**Peak Area (%)**
**PLA-1** (NaOH)	28400	1.35	8.74
	360	1.08	31.26
	90	1.08	60
**PCL-1** (NaOH)	4480	1.17	32.80
	1620	1.09	38.33
	370	1.02	4.27
	100	1.02	24.60
**PVL-1** (NaOH)	64860	1.06	2.66
	830	1.61	97.34
**PLA-1** (DBU)	240	1.14	100
**PCL-1** (DBU)	2200	4.3	100
**PVL-1** (DBU)	2100	2.33	89.04
	420	1.00	4.09
	260	1.00	6.87

M_n values were determined using an Agilent 1260 Infinity II Multi-Detector GPC system through PLgel 5 µm columns packed with PSDVB beads. Samples were run in THF at a flow rate of 1 mL min^{-1} at 35 °C. Molecular weights were subsequently determined using RI detector based on a calibration obtained using narrow dispersity polystyrene standards.

References:

1. Hormnirun, P.; Marshall, E. L.; Gibson, V. C.; Pugh, R. I.; White, A. J. P. Study of Ligand Substituent Effects on the Rate and Stereoselectivity of Lactide Polymerization Using Aluminum Salen-Type Initiators. *Proc. Natl. Acad. Sci. U. S. A.* **2006**, *103*, 15343–15348.
2. Brutman, J. P.; Delgado, P. A.; Hillmyer, M. A. Polylactide Vitrimers. *ACS Macro Lett.* **2014**, *3*, 607–610.
3. *Poly(caprolactone)*. https://polymerdatabase.com/polymers/polycaprolactone.html (accessed 2022-11-28).
4. Furuhashi, Y.; Sikorski, P.; Atkins, E.; Iwata, T.; Doi, Y. Structure and Morphology of the Aliphatic Polyester Poly(δ-Valerolactone) in Solution-Grown, Chain-Folded Lamellar Crystals. *J Polym Sci B Polym Phys* **2001**, *39*, 2622–2634.
